# Evidence for a serpentinized plate interface favouring continental subduction

**DOI:** 10.1038/s41467-020-15904-7

**Published:** 2020-05-01

**Authors:** Liang Zhao, Marco G. Malusà, Huaiyu Yuan, Anne Paul, Stéphane Guillot, Yang Lu, Laurent Stehly, Stefano Solarino, Elena Eva, Gang Lu, Thomas Bodin, CIFALPS Group, Liang Zhao, Marco G. Malusà, Anne Paul, Stéphane Guillot, Stefano Solarino, Elena Eva, Gang Lu, AlpArray Working Group, Anne Paul, Stefano Solarino

**Affiliations:** 1 State Key Laboratory of Lithospheric Evolution, Institute of Geology and Geophysics, Chinese Academy of Sciences, Beijing, China; 2 Department of Earth and Environmental Sciences, University of Milano-Bicocca, Milan, Italy; 3 Istituto Nazionale di Geofisica e Vulcanologia, ONT, Genova, Italy; 4 ARC Centre of Excellence for Core to Crust Fluids Systems, Department of Earth and Environmental Sciences, Macquarie University, North Ryde, Australia; 5 Centre for Exploration Targeting, University of Western Australia, Perth, Australia; 6 Geological Survey of Western Australia, Perth, Australia; 7 Univ. Grenoble Alpes, Univ. Savoie Mont Blanc, CNRS, IRD, IFSTTAR, ISTerre, 38000 Grenoble, France; 8 Univ. Lyon, Universite Lyon 1, Ens de Lyon, CNRS, UMR 5276 LGL-TPE, F-69622 Villeurbanne, France

**Keywords:** Geodynamics, Seismology, Tectonics

## Abstract

The dynamics of continental subduction is largely controlled by the rheological properties of rocks involved along the subduction channel. Serpentinites have low viscosity at geological strain rates. However, compelling geophysical evidence of a serpentinite channel during continental subduction is still lacking. Here we show that anomalously low shear-wave seismic velocities are found beneath the Western Alps, along the plate interface between the European slab and the overlying Adriatic mantle. We propose that these seismic velocities indicate the stacked remnants of a weak fossilised serpentinite channel, which includes both slivers of abyssal serpentinite formed at the ocean floor and mantle-wedge serpentinite formed by fluid release from the subducting slab. Our results suggest that this serpentinized plate interface may have favoured the subduction of continental crust into the upper mantle and the formation/exhumation of ultra-high pressure metamorphic rocks, providing new constraints to develop the conceptual and quantitative understanding of continental-subduction dynamics.

## Abstract (web summary)

The dynamics of continental subduction is largely controlled by the rheological properties of rocks involved along the subduction channel. Here, the authors reveal a prominent, yet previously undetected, low-velocity body beneath the Western Alps, along the plate interface between the European slab and the overlying Adriatic mantle, which they interpret as a serpentinite layer.

## Introduction

Lithosphere rheology is a key parameter to understand the dynamics of continental subduction following the closure of paleo-oceans during convergence between tectonic plates. Serpentinites are commonly inferred in many subduction zones^1–3^. They are the principal water carrier toward the mantle, with effective viscosity several orders of magnitude lower than that of the dry upper mantle at geological strain rates^4^. Due to these unique properties, serpentinites are expected to play a major role in controlling the bulk rheology of subduction zones^5^.

**Fig. 1:**
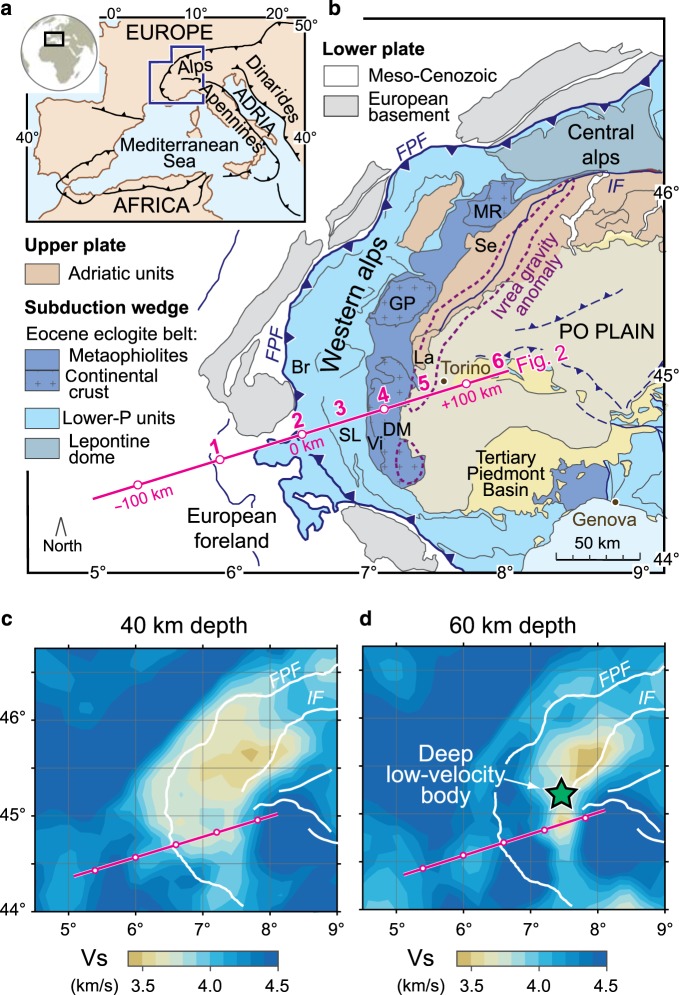
Geologic setting and depth slices of Vs. **a** Tectonic sketch map of the Adria–Europe plate boundary zone. **b** Geological sketch map of the western Alpine region (modified from Malusà et al.^45^). The magenta line indicates the location of the cross section of Fig. 2a. Numbers 1–6 indicate the sites of the depth-velocity profiles illustrated in Fig. 2b. The purple line indicates the gravity anomaly of the Ivrea body (0 mGal isoline). Acronyms: Br, Briançonnais; DM, Dora-Maira; FPF, Frontal Pennine Fault; GP, Gran Paradiso; IF, Insubric Fault; La, Lanzo; MR, Monte Rosa; Se, Sesia-Lanzo; SL, Schistes lustrés; Vi, Viso. **c**, **d** Vs structure at different depths (40 km and 60 km) after TransD inversion. The green star marks the deep–low-velocity body (yellow to wheat area).

The Western Alps is one of the best-preserved subduction wedges in the world^6,7^ (Fig. 1). They are the result of Cretaceous-to-Paleogene subduction of the Tethys Ocean and adjoining European continental margin beneath the Adriatic microplate^8^. The Alpine subduction wedge includes abundant high-pressure serpentinites^9–11^ mainly exposed above (ultra)high-pressure (UHP) gneissic domes characterized by mineral assemblages attesting to continental subduction^12,13^ (Dora-Maira, Gran Paradiso, and Monte Rosa in Fig. 1b). The deep configuration of this subduction wedge is increasingly well documented by geophysical data^14–22^, but a high-resolution image of seismic velocity structure for the deepest levels of the plate interface is still missing. As a consequence, potential rheology variations within the subduction channel remain largely unconstrained, which precludes a full understanding of the dynamics of crucial stages of continental subduction and exhumation of UHP rocks.

Laboratory experiments show that serpentinization significantly reduces seismic velocity and density of the precursor peridotite^23,24^, thus providing a viable approach for probing the distribution of serpentinites within a subduction zone. However, based on numerical models, the sustainable thickness of a serpentinized channel may be on the order of a few kilometers only^25^, i.e., close to the limit of seismic detectability^26^. This implies that seismic probing requires cutting-edge imaging techniques.

Here, we apply a Bayesian transdimensional (TransD) inversion technique^27–29^ to invert Rayleigh wave group velocity dispersion data (Supplementary Note 1 and Supplementary Figs. 1–7) for a shallow upper mantle shear-wave velocity model in the Western Alps. The inversion treats the model parameterization (e.g., number of velocity layers) as an unknown in the inversion to be determined by data. This avoids regularizing the inverse problem and yields more robust velocity amplitude estimates^27^. The tomography is conducted from dispersion data^19^ measured in the area of the first densely spaced array of broadband seismic stations across the Western Alps (Fig. 1)^15^ (Methods and Supplementary Note 1). This approach provides absolute shear-wave velocity (Vs) information (Supplementary Figs. 8–14) and an unprecedented high-resolution image of the velocity structure at the plate interface of a continental subduction zone.

## Results and discussion

### Geophysical evidence of a serpentinized subduction channel

**Fig. 2:**
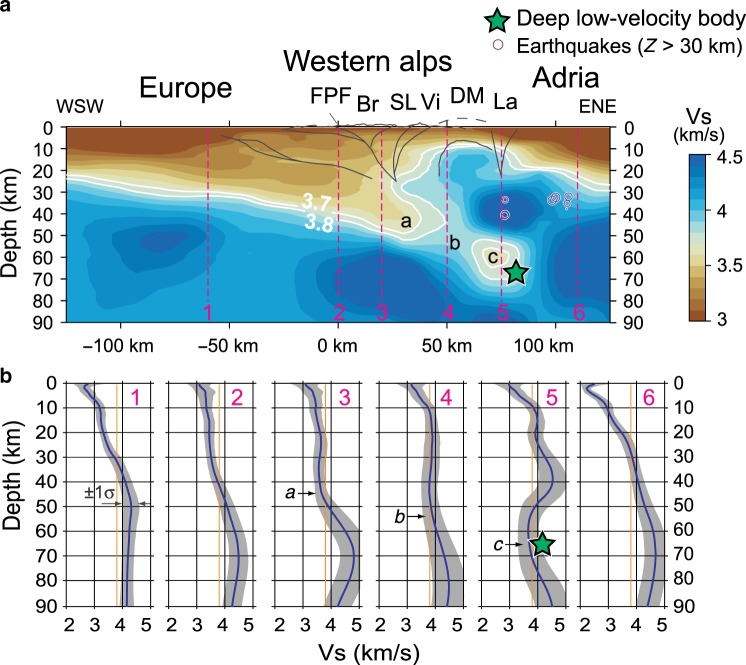
Shear-wave velocity model across the Western Alps. **a** Cross-section showing the absolute Vs in the 0–90-km depth range (see location in Fig. 1b). The white lines indicate the 3.7 and 3.8 km s^−1^ contours. The green star marks the deep–low-velocity body. Earthquake foci (depth > 30 km) in a 40-km-wide swath parallel to the profile are plotted as purple circles. Letters a–c indicate regions of the model discussed in the text. The main crustal features (black lines) after Zhao et al.^15^ and Malusà et al.^17^ (acronyms as in Fig. 1b). **b** Depth–velocity profiles (±1*σ*) beneath six typical sites along the transect (see location in Fig. 1b and panel **a**). The orange vertical line is the 3.8 km s^−1^ isovelocity.

Figure 2a shows a cross-section of the inverted velocity model across the southern Western Alps down to 90-km depth, and the main tectonic features, indicated by black lines, based on available geologic constraints^14,17,30^. At shallow depth, the inverted velocity model shows low Vs regions corresponding to the main sedimentary basins formed on European and Adriatic crusts (Southeast basin and Po Plain, respectively). The European lithospheric mantle (in blue in Fig. 2) is clearly underthrusted beneath the Adriatic mantle imaged on the eastern part of the profile. In general, the subduction wedge beneath the Western Alps exhibits a normal velocity structure with Vs progressively increasing with depth, from ~3.3 km s^−1^ in the uppermost levels, to ~3.6 km s^−1^ (wheat colors) at ~40-km depth (profile 3 in Fig. 2a). On the Adriatic side of the cross section (around +60-km distance, between profiles 4 and 5), the culmination of the region with Vs >4.0 km s^−1^ at ~10–15-km depth matches with the Ivrea gravity anomaly (Fig. 1b; see also Supplementary Fig. 8), and with the high-velocity regions beneath the Dora-Maira dome independently observed in receiver function^15^ and local earthquake tomography models^21^ (see also Supplementary Figs. 15–17).

The most intriguing feature of the inverted model is the low-velocity region with Vs ≤ 3.7 km s^−1^ imaged between 50- and 70-km depth and around +75-km distance (profile 5, green star in Fig. 2a). This low-velocity region, squeezed between the European lithospheric mantle and the overlying Adriatic mantle, may mark the fossil subduction channel beneath the Western Alps. Along this channel, Vs initially increases from ~3.6 km s^−1^ at ~45-km depth (region a in Fig. 2a) to ~3.9 km s^−1^ at ~55-km depth (region b in Fig. 2a), and then decreases again to ~3.6 km s^−1^ down to ~70-km depth (region c in Fig. 2a). The distinctive velocity structure of region c is even more evident in the depth-velocity profiles reported in Fig. 2b, where the blue lines and associated gray bands indicate the velocity and associated 1*σ* error after the TransD inversion. All of these profiles show crustal velocities increasing with depth, apart from profile 5 that shows a major velocity drop at ~45-km depth. This “inverted” crustal velocity structure agrees well with previous receiver function results showing a negative velocity gradient beneath the Dora-Maira^15^ (see also Supplementary Fig. 17).

The lateral extent of the deep–low-velocity region can be evaluated in the horizontal slices of Fig. 1c, d. As shown in Fig. 1d, the region with Vs < 3.7 km s^−1^ is observed, at 60-km depth, all along the arc of the Western Alps, from the Dora-Maira to the Monte Rosa domes (DM and MR, respectively, in Fig. 1b). This attests to a clear linkage between the Eocene eclogite belt, which is part of the Alpine subduction wedge, and the deep–low-velocity region highlighted by TransD images. Similar to Fig. 2, two additional cross sections are presented in Supplementary Figs. 10 and 11.

**Fig. 3:**
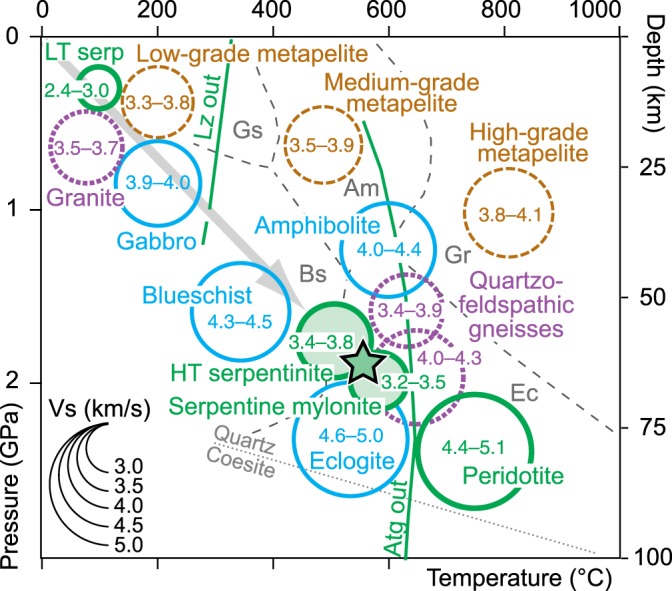
Measured Vs (km s^−1^) for different rocks (green = ultramafic; blue = mafic; purple = granitic; brown = pelitic) at ambient conditions. The size of the circles is proportional to Vs, while circle location depends on the pressure–temperature range at equilibrium for each rock type^23,31–39,56^. The Vs range of 4.0–4.3 km s^−1^ in quartzofeldspathic gneisses is based on calculations for modeled rocks at 700 °C and 2 GPa^36^. The lower bound for HT serpentinite (3.4 km s^−1^) is calculated from single-crystal elastic data and referred to Reuss approximation and 2 GPa^23^. Lizardite (Lz) and antigorite (Atg) stability fields^57,58^. The dashed gray lines indicate the metamorphic facies boundaries (Am amphibolite, Bs blueschist, Ec eclogite, Gr granulite, Gs greenschist). Note the progressive increase in Vs with depth and metamorphic grade in pelitic, granitic, and mafic rocks, and the sharp change in Vs across the Atg-out curve in ultramafic rocks. The green star marks the rock types consistent with Vs and depth of the analyzed low-velocity body.

The geologic interpretation of the peculiar velocity structure beneath the Alpine wedge requires independent knowledge of the Vs ranges for the different rock types likely involved in the Alpine subduction zone. These Vs ranges are provided by a number of laboratory experiments^23,31–33^ that are summarized in Fig. 3.

Based on the composition of the oceanic slivers now exposed in the Alpine belt, various proportions of serpentinized mantle, basalts, and sedimentary rocks were subducted at the Alpine trench since the Cretaceous, during the oceanic subduction stage. Subsequent Paleogene continental subduction additionally involved granitic and (meta)sedimentary rocks from the hyperextended European margin. All of these rocks display a progressive increase in Vs with depth and metamorphic grade, which is particularly evident in pelitic, granitic, or mafic rocks (Fig. 3). For a given rock type, the effects of increasing pressure with depth, which would imply an increase in Vs, are partly compensated by the effects of increasing temperature that would imply a Vs decrease. As a result, major changes in Vs with depth are mainly due to changes in metamorphic mineral assemblages.

In metapelites, Vs values provided by laboratory experiments progressively increase from 3.3 to 3.8 km s^−1^ in low-grade metapelites^34^ to 3.8–4.1 km s^−1^ in high-grade metapelites^32^. In mafic rocks, Vs values progressively increase from 3.9 to 4.0 km s^−1^ in gabbros to 4.3–4.5 km s^−1^ in blueschists to 4.6–5.0 km s^−1^ in mafic eclogites^33^. Vs values of 3.5–3.7 km s^−1^ measured in granites^35^ are expected to increase up to 4.0–4.3 km s^−1^ in quartzofeldspathic gneisses under eclogite facies conditions^36^. In fully hydrated ultramafic rocks, measured Vs values increase from 2.4 to 3.0 km s^−^^1^ in low-temperature lizardite serpentinite^37^ to 3.4–3.8 km s^−1^ in high-temperature antigorite serpentinite^23,37^, but Vs as low as 3.2 km s^−1^ could be observed in antigorite mylonites due to the platy shape of antigorite crystals and the related strong fabric^38^. A sharp change in Vs, toward values of 4.4–5.1 km s^−1^ that are typical of peridotite, is however expected across the antigorite-out curve^32,33,39^, with Vs linearly increasing for decreasing volume fraction of antigorite^37,40^ (Fig. 3).

The normal velocity structure of the Alpine subduction wedge down to 50 km is fully consistent with the Vs values, progressively increasing with depth, measured in metapelites and gneisses that are exposed in UHP gneissic domes at the Earth surface. By contrast, the progressive Vs decrease from region b to region c within the subduction channel (Fig. 2a) cannot be explained by metamorphic phase changes in metapelites and gneisses. The only rock characterized by Vs values of ~3.6 km s^−1^ in the 55–70-km depth range is serpentinite. We propose that Fig. 2a may provide evidence of a weak serpentinite channel preserved in the 55–70-km depth range along the plate interface (Methods). Alternatively, the low Vs values in region c may be due to high pore-fluid pressures, which would in turn promote ongoing serpentinization. Serpentinization favors aseismic creep^4^, in line with the aseismic character of the deep–low-velocity body (Fig. 2a). At greater depth, the sharp increase in Vs along the subduction channel attests to the destabilization of antigorite and transformation of serpentinite into peridotite.

### Progressive development of the serpentinized plate interface

**Fig. 4:**
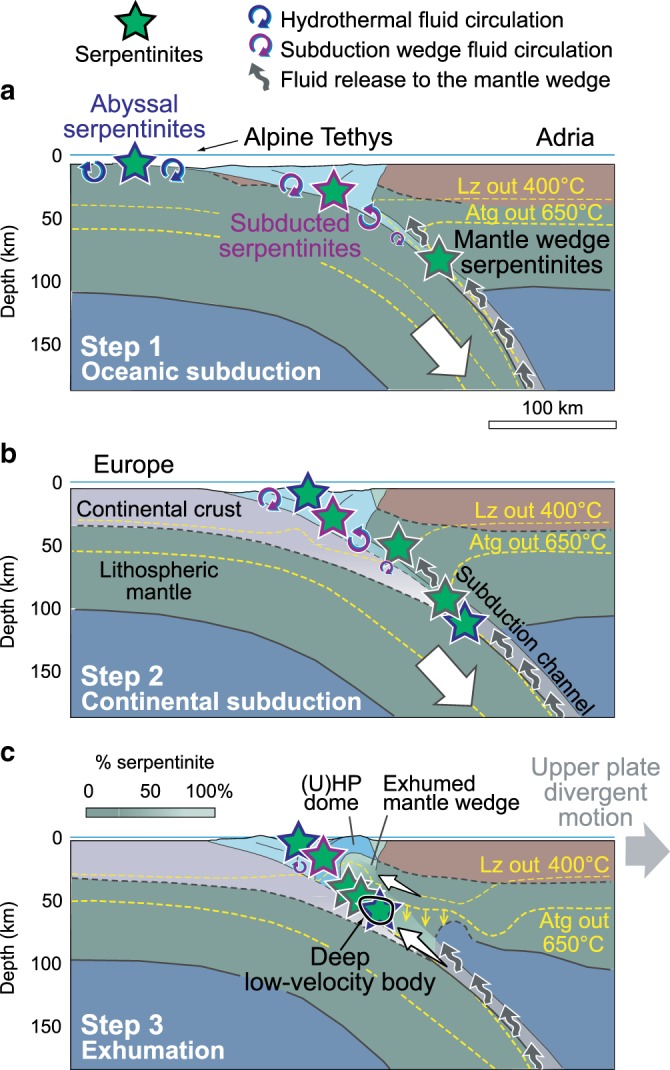
Environments of serpentinization during subduction and geologic interpretation of the deep–low-velocity body. **a** Oceanic subduction. **b** Continental subduction. **c** Upper-plate divergent motion and exhumation of UHP rocks (crustal features color coded as in Fig. 1b). Lithospheric structure after Zhao et al.^14,15^, Lyu et al.^16^, and Solarino et al.^21^. Environments of serpentinization and fluid circulation after Deschamps et al.^41^. Evolution of lizardite (Lz) and antigorite (Atg) destabilization isotherms after Liao et al.^46^. Percentage of serpentinite in the exhumed mantle wedge after Solarino et al.^21^. The deep–low-velocity body imaged in the subduction channel consists of abyssal and mantle-wedge serpentinites that lubricate the plate interface and facilitate continental subduction.

Serpentinite preserved along the continental subduction channel may have formed by three different processes in different settings (Fig. 4), including hydration of oceanic peridotite by seafloor hydrothermal activity/seawater alteration (abyssal serpentinite), fluids released from the subducted slab to the overlying mantle wedge (mantle-wedge serpentinite), and fluids circulating and affecting ultramafic rocks within the subduction wedge (subducted serpentinite)^41^.

Abyssal serpentinite formed when the Tethys ocean was still open, whereas mantle-wedge serpentinites formed by fluid release during oceanic subduction (Fig. 4a). In oceanic domains characterized by slow spreading rates as in the Western Alps, serpentinization may have occurred over a thickness of 2–4 km reaching extents of 70–80%^42^. During subsequent continental subduction, these abyssal serpentinites were either stacked in the subduction wedge, or dragged toward the subduction channel together with basalts and sediments of the Tethys seafloor and rocks of the European paleomargin (Fig. 4b). An example of such stacking is provided by the 10-km-thick Viso unit (Vi in Fig. 1), which includes different slivers of serpentinites with eclogites^6^. During subduction, meta-ophiolites experienced breakdown of amphibole and zoisite, and partial dehydration of antigorite that was likely completed at ~180-km depth along the cold Alpine geothermal gradient (<7 °C km^−1^)^6,43,44^. Continental rocks experienced progressive dehydration breakdown of zoisite, chlorite, biotite, talc, and lawsonite, but the low geothermal gradient of the Alpine subduction zone prevented the breakdown of phengite in UHP continental rocks. Dehydration of metasediments and mafic rocks was likely completed at 200–250 km^44^. Substantial amounts of aqueous fluids were thus released at sub-arc depths (80–180 km), promoting further serpentinization both in the subduction wedge and along the plate interface above the subduction channel. Minor amounts of fluids are possibly released from the slab even today, despite the fact that subduction is no longer active, thus promoting further serpentinization and lowering seismic velocities^44^.

We propose that the resulting serpentinized plate interface made of subducted serpentinites and mantle wedge serpentinites may have controlled the bulk rheology of the Alpine subduction zone during the Cenozoic, facilitating the subduction of European continental lithosphere into the Adriatic upper mantle, and enhanced the formation of UHP metamorphic rocks that are now exposed in the Dora-Maira dome. During fast exhumation of the Dora-Maira rocks in the Late Eocene, which was possibly triggered by a component of divergent motion of the upper plate^7,45,46^, subducted abyssal serpentinites and the adjoining mantle-wedge serpentinites raised along the subduction channel (Fig. 4c). Numerical models^46^ suggest that the subduction channel may become much thicker after upper-plate divergent motion compared with the previous stages of plate convergence, and may include slivers of serpentinite formed in different settings. Moreover, the subduction channel is squeezed between the two plates during the transition from continental subduction to collision favoring its thickening. Numerical models also predict that the transient uplift of isothermal surfaces during fast exhumation is rapidly followed by thermal relaxation. In the Western Alps, this likely allowed the preservation of a thick composite volume of partly serpentinized mantle rocks down to depths of ~70 km that can be now detected by cutting-edge tomographic methods. However, the precise values of thickness and the amount of serpentinization of this composite rock volume remain uncertain due to the trade-off between them (Methods). Our observations may provide an ideal example for developing conceptual fameworks of continental subduction, and then have the potential of rendering more accurate dynamic modeling and comparisons with multidisciplinary evidence.

## Methods

In this work, we apply a Bayesian transdimensional (TransD) tomography technique^27,28,47^ to invert a high-quality Rayleigh wave dispersion dataset^19^ for crustal and upper mantle shear-wave velocity structure in the Western Alps. The TransD inversion treats the number of velocity layers as an unknown parameter that is determined in the inversion^48^. This avoids subjective choice in the inversion, such as manual tuning of damping and regularization parameters. The technique is capable of retrieving high-resolution velocity models of the crust and uppermost mantle^29,49,50^.

### Dispersion dataset and transdimensional inversion

The dispersion dataset^19^ takes advantage of the large number of seismic stations in Europe, in particular in the great Alpine region with the high-density AlpArray and CIFALPS temporary experiments. This dataset provides the highest possible data coverage (0.15° grid spacing) in our study area. At each geographical location, we carry out a 1D inversion of the local dispersion curve, and invert for Vs perturbations around a reference model. The reference model is set to a homogeneous half-space with no discontinuities, and the a priori distribution for velocity variations is set to a uniform distribution (3.8 km s^−1^) with a relatively wide range (±50%). The latter is the major difference compared with the Bayesian inversion^19^ that imposed a monotonous velocity increase with depth, and therefore could not detect the low-velocity zones featured in this study. Finally, the set of 1D models computed at each location are combined to produce a laterally varying 3D velocity model (more details in Supplementary Note 1).

We formulate the inverse problem in a Bayesian framework where all information is represented in probabilistic terms. The solution of the problem is the posterior probability distribution that represents the probability density of the model parameter Vs. We use the reversible-jump Monte Carlo algorithm^51^ to generate samples from the posterior distribution. At each iteration of the algorithm, a new 1D velocity model is proposed and its estimated dispersion curve is compared with observations. The proposed model is then either accepted or rejected in the ensemble solution. After a large number of iterations, the ensemble of accepted models is used to approximate the posterior distribution. The algorithm prevents overfitting the data by penalizing overcomplex solution models^27^. Uncertainties in the solution and trade-offs between model parameters are evaluated using the posterior model variance and covariance. For a detailed description of the model parameterization, the algorithm, and tomographic inversion, as well as model robustness and effects of the anisotropic serpentinite layer, we refer the reader to methodology papers^27,29^ and to Supplementary Figs. 18–20.

### Model robustness

To illustrate the robustness of the inverted shear-wave velocity model, we first compare our inversion results with the Vs model^19^ along the three cross sections (Supplementary Figs. 12–14). Both studies use the same dispersion maps for the final 3D velocity model, but our TransD inversion allows negative velocity gradient with respect to depth (i.e., low-velocity zones). We compare misfit between the data and predicted dispersions of the two models (Supplementary Figs. 7 to 9, panels b and c), and a form of data variance reduction (panel d):

$$\left( {1 - \frac{{\rm{variance}\left( {data - synthetics} \right)}}{{\rm{variance}\left( {data} \right)}}} \right) \times 100.$$

Here synthetics are the predicted group velocities for the two models. Our new model shows in general a better fit over the previous Vs model^19^ across the low-velocity zone described in the text.

We further compare the velocity features with other available seismic models along the CIFALPS and ECORS-CROP^52^ lines (Supplementary Figs. 15–17). Our model shows many consistent crustal features, such as the high-velocity Ivrea body and the low-velocity basins, that are spatially comparable with higher-resolution models (Supplementary Figs. 15 and 16).

In Supplementary Fig. 17, we compare the receiver function common-conversion-point (CCP) stacked model^15^ along the CIFALPS line with the depth gradient of the inverted velocity model. The depth gradient was computed with 0.25-km vertical spacing. This comparison shows that both the CCP section and the Vs model detect the Moho of the subducted European lithosphere, and most interestingly, the top boundary of the low-velocity zone. Although the amplitude of P-to-S-converted phases at velocity boundaries mapped with the CCP section cannot be simply explained by vertical velocity gradients, the correspondence between Supplementary Figs. 17a and b in the distance range 50–80 km is striking. In Supplementary Fig. 17a, the deep Ps phase at 70–75-km depth, 60–80-km distance that was interpreted as the deepest trace of the European Moho ever imaged in the Western Alps^15^, well coincides with the strong positive Vs gradient at the same location in Supplementary Fig. 17b. A similar coincidence is also striking on top of the LVZ at 45–50-km depth (the same 60–80-km distance range) with an “inverted” Moho of negative Ps amplitude in Supplementary Fig. 17a and a negative velocity gradient in Supplementary Fig. 17b. However, the strong negative Ps phase at 20–35-km depth and 25–70-km distance in Supplementary Fig. 17a is not that well explained by a strong negative velocity gradient in Supplementary Fig. 17b, showing the limits of this comparison. Nevertheless, we think that the discovery of the low-velocity deep body at the subduction interface from the TransD inversion of group velocity dispersion data, which compares well with the independent CCP image may be attributed to the high resolution of the dispersion dataset associated with the high station density along the CIFALPS line.

### Effects of slow-velocity layer and seismic anisotropy

We address here the question of the effective thickness of the slow-velocity serpentinite layer recoverable by our dispersion dataset. We also test the influence of the anisotropy of the serpentinite layer in our isotropic two-step tomographic inversion.

*Recovery of layer thickness*: We construct simple 1D layered models that include a slow-velocity layer whose top is at 40-km depth with 10-, 15-, 20-, 25-, and 30-km layer thickness, respectively. Synthetic group velocities are computed, and randomly distributed Gaussian noise with ~100 m s^−1^ standard deviation is added. We then carry out the transdimensional inversion with the same uniform prior model used for the real data inversions. The modeling results are presented in Supplementary Fig. 18. The inversion tests show that the inversions can recognize a slow-velocity input layer, but the amplitude recovery is not satisfactory for layer thicknesses smaller than 15 km. The exact depth of the velocity discontinuities is less well recovered because surface wave dispersion data are not very sensitive to velocity gradient. It has been shown that by incorporating a smooth 1D background model with discontinuity depth inferred from receiver functions, the transdimensional inversion may better recover the velocity jump at a discontinuity^29^. The synthetic tests shown here, however, do not use this approach^29^ to be consistent with the inversion of real data used in this paper.

*Effects of an anisotropic layer on the isotropic inversion*: Serpentinites are highly anisotropic rocks with a slow-velocity axis of hexagonal symmetry^53^. Our two-step tomographic inversion, i.e., the group velocity map inversion^19^ and the transdimensional inversion conducted here, is isotropic only. It is well-known that the presence of seismic anisotropy may cause artifacts in tomographic inversions^54^. However, the evaluation of the full 3D effects of such an anisotropic layer in noise tomographic inversion may require numerical simulations of noise correlations, which are beyond the scope of this study.

Here we take a simple approach by inverting back-azimuthally dependent synthetic dispersion data for an isotropic 1D velocity profile to assess whether imaging artifacts may be raised by our isotropic tomographic approach. We assume that anisotropy in the serpentinite layer may be simulated by a slow velocity with hexagonal symmetry; the effect of anisotropy in the dipping layer may be approximated by that of a horizontal layer with dipping symmetry axis; the anisotropic signal used for the transdimensional inversion is effectively smoothed by the group velocity inversion, especially in the western Alps where the azimuthal distribution of interstation paths is optimum thanks to the array geometry.

We use the anisotropic reflectivity package ANIPROP^55^ to compute the group velocities at the same periods as in the real data. To maximize the anisotropy effects, we take the 30-km low-velocity layer model of the previous thickness tests. The strength of anisotropy is fixed at −8%^53,55^. We compute the dispersion curves from 0 to 350° back-azimuths with 10° increments; then we average to obtain the effective group velocity dispersion curve and inverted it to obtain the 1D velocity profile. This procedure assumes that the isotropic ambient noise group velocity inversion has averaged the anisotropic signal over the range of back-azimuths. The inverted velocity model is then compared with the input model for the anisotropic effects.

We test two models, including a slow-velocity layer with horizontal and 45° tilted symmetry axis; for comparison, we also include the fast-velocity symmetry axis cases. As shown in Supplementary Fig. 19, individual Rayleigh wave dispersion curves respond to different incident back-azimuths in both the fast- and slow-velocity horizontal symmetry axis cases. Their average is very close to the dispersion computed from the isotropic velocity model with the same 30-km slow-velocity thickness. The inverted velocity models well recover the slow velocity of the input model. The horizontal fast symmetry axis model slightly pushes down the bottom of the velocity layer. The effect of anisotropy is maximum in the tilted symmetry axis examples. In the case of a tilted slow-velocity symmetry axis, the recovered isotropic slow velocity is overestimated (~0.1 km s^−1^), while the recovered layer thickness is larger (~5 km) than in the average case. For the tilted fast-velocity symmetry axis case, the recovered amplitude of the layer is underestimated, and the recovered layer is slightly thinner than in the averaged case.

In conclusion, in the presence of a thick tilted serpentinite layer, anisotropy in the dispersion signal may cause the isotropic inversion to overestimate both the slow-velocity amplitude and the layer thickness. Considering the results from thickness tests, we suggest that our isotropic inversions may be capable of revealing a 20- to 25-km-thick slow-velocity (~3.7–3.8 km s^−1^) layer; however, the amplitude of the slow-velocity layer remains uncertain.

*Velocity of the serpentinite layer*: To further illustrate the robustness of the velocity estimate of the serpentinite layer in the preferred model, we operate two more tests using a model with larger variations in the lower-velocity channel, and a homogeneous model of 4.0 km s^−1^ from 10 km downward (Supplementary Fig. 20). These are compared with the final model at Location 5 along the CIFALPS line. As shown in Supplementary Fig. 20a, both test models lie pretty much within the 1*σ* model uncertain area, and the predicted dispersion curves are close to those of the final model (Supplementary Fig. 20b). However, the misfit distribution of these two test models is not centered, and the 4 km s^−1^ homogeneous model even shows a bimodal distribution. This implies that these two test models provide a poorer fit to the data than the final model.

In addition, the error at a given depth is not independent from the error at another depth. For example, an increase of Vs at 40 km needs a decrease in Vs at 65 km, and vice versa. Our previous receiver function results^15^ indicate a strong upward velocity increase attested by a strong negative energy of P-to-S conversion (Supplementary Fig. 17). Therefore, we can preclude an increase of Vs at 65 km; otherwise, a decrease of Vs at 40 km would give a smooth discontinuity. The other possibility, to the extreme, is having Vs = 5 km s^−1^ at 40 km to get 3.3 km s^−1^ at 65 km to fit the data, but this is very unlikely because even 100% serpentinite cannot satisfy this low-velocity value of 3.3 km s^−1^.

## Supplementary information

Supplementary Information

Description of Additional Supplementary Files

Supplementary Data 1

## Data Availability

The authors declare that the data supporting the findings of this study are available within the paper and its Supplementary information file (Supplementary Data 1).

## Code sharing

Software package of Bayesian Transdimensional tomography technique^27,28,46^ is available, and may be accessible through coauthor (T.B., email: Thomas.bodin@ens-lyon.fr or H.Y., email: huaiyu.yuan@mq.edu.au).

## References

[1] Abers GA (2005). Seismic low-velocity layer at the top of subducting slabs: observations, predictions, and systematics. Phys. Earth Planet..

[2] Bostock MG, Hyndman RD, Rondenay S, Peacock SM (2002). An inverted continental Moho and serpentinization of the forearc mantle. Nature.

[3] Kawakatsu H, Watada S (2007). Seismic evidence for deep-water transportation in the mantle. Science.

[4] Hilairet, N. et al. High-pressure creep of serpentine, interseismic deformation, and initiation of subduction. *Science* **318** (2007). 10.1126/science.1148494 18096804

[5] Guillot S, Schwartz S, Reynard B, Agard P, Prigent C (2015). Tectonic significance of serpentinites. Tectonophysics.

[6] Guillot S, Hattori K, Agard P, Schwartz S, Vidal. O (2009). Exhumation Processes in Oceanic and Continental Subduction Contexts: A Review.

[7] Malusà MG, Faccenna C, Garzanti E, Polino R (2011). Divergence in subduction zones and exhumation of high-pressure rocks (Eocene Western Alps). Earth Planet. Sci. Lett..

[8] Handy MR, Schmid SM, Bousquet R, Kissling E, Bernoulli D (2010). Reconciling plate-tectonic reconstructions of Alpine Tethys with the geological–geophysical record of spreading and subduction in the Alps. Earth Sci. Rev..

[9] Scambelluri M, Strating EHH, Piccardo GB, Vissers RLM, Rampone E (1991). Alpine olivine- and titanian clinohumite-bearing assemblages in the Erro-Tobbio peridotite (Voltri Massif, NW Italy). J. Metamorph. Geol..

[10] Schwartz S (2013). Pressure-temperature estimates of the lizardite/antigorite transition in high pressure serpentinites. Lithos.

[11] Cannaò E, Scambelluri M, Agostini S, Tonarini S, Godard M (2016). Linking serpentinite geochemistry with tectonic evolution at the subduction plate-interface: The Voltri Massif case study (Ligurian Western Alps, Italy). Geochim. Cosmochim. Ac..

[12] Chopin C (1984). Coesite and pure pyrope in high-grade blueschists of the Western Alps: a first record and some consequences. Contrib. Mineral. Petr..

[13] Rubatto D, Hermann J (2001). Exhumation as fast as subduction?. Geology.

[14] Zhao L (2016). Continuity of the Alpine slab unraveled by high-resolution P-wave tomography. J. Geophys. Res..

[15] Zhao L (2015). First seismic evidence for continental subduction beneath the Western Alps. Geology.

[16] Lyu C, Pedersen HA, Paul A, Zhao L, Solarino S (2017). Shear wave velocities in the upper mantle of the Western Alps: new constraints using array analysis of seismic surface waves. Geophys. J. Int..

[17] Malusà MG (2017). Earthquakes in the western alpine mantle wedge. Gondwana Res..

[18] Beller S (2018). Lithospheric architecture of the South-Western Alps revealed by multiparameter teleseismic full-waveform inversion. Geophys. J. Int..

[19] Lu Y, Stehly L, Paul A, AlpArray Working Group. (2018). High-resolution surface wave tomography of the European crust and uppermost mantle from ambient seismic noise. Geophys. J. Int..

[20] Salimbeni S (2018). Active and fossil mantle flows in the western Alpine region unravelled by seismic anisotropy analysis and high-resolution P wave tomography. Tectonophysics.

[21] Solarino S (2018). Mantle wedge exhumation beneath the Dora-Maira (U)HP dome unravelled by local earthquake tomography (Western Alps). Lithos.

[22] Sun W, Zhao L, Malusà MG, Guillot S, Fu LY (2019). 3-D Pn tomography reveals continental subduction at the boundaries of the Adriatic microplate in the absence of a precursor oceanic slab. Earth Planet. Sci. Lett..

[23] Bezacier L, Reynard B, Cardon H, Montagnac G, Bass JD (2013). High‐pressure elasticity of serpentine and seismic properties of the hydrated mantle wedge. J. Geophys. Res..

[24] Reynard B (2013). Serpentine in active subduction zones. Lithos.

[25] Schwartz S, Allemand P, Guillot S (2001). Numerical model of the effect of serpentinites on the exhumation of eclogitic rocks: insights from the Monviso ophiolitic massif (Western Alps). Tectonophysics.

[26] Hilairet N, Reynard B (2009). Stability and dynamics of serpentinite layer in subduction zone. Tectonophysics.

[27] Bodin T, Sambridge M, Rawlinson N, Arroucau P (2012). Transdimensional tomography with unknown data noise. Geophys. J. Int..

[28] Bodin T (2012). Transdimensional inversion of receiver functions and surface wave dispersion. J. Geophys. Res..

[29] Yuan H, Bodin T (2018). A probabilistic shear wave velocity model of the crust in the central West Australian Craton constrained by transdimensional inversion of ambient noise dispersion. Tectonics.

[30] Lardeaux JM (2006). A crustal‐scale cross‐section of the south‐western Alps combining geophysical and geological imagery. Terra Nova.

[31] Weiss T, Siegesmund S, Rabbel W, Bohlen T, Pohl M (1999). Seismic velocities and anisotropy of the lower continental crust: a review. Pure Appl. Geophys..

[32] Khazanehdari J, Rutter EH, Brodie KH (2000). High‐pressure‐high‐temperature seismic velocity structure of the midcrustal and lower crustal rocks of the Ivrea‐Verbano zone and Serie dei Laghi, NW Italy. J. Geophys. Res..

[33] Bezacier L, Reynard B, Bass JD, Wang J, Mainprice D (2010). Elasticity of glaucophane, seismic velocities and anisotropy of the subducted oceanic crust. Tectonophysics.

[34] Ji S (2015). Magnitude and symmetry of seismic anisotropy in mica‐and amphibole‐bearing metamorphic rocks and implications for tectonic interpretation of seismic data from the southeast Tibetan Plateau. J. Geophys. Res..

[35] Rudnick RL, Fountain DM (1995). Nature and composition of the continental crust: a lower crustal perspective. Rev. Gophys..

[36] Brownlee SJ (2011). Predicted velocity and density structure of the exhuming Papua New Guinea ultrahigh‐pressure terrane. J. Geophys. Res..

[37] Ji S (2013). Seismic velocities, anisotropy, and shear‐wave splitting of antigorite serpentinites and tectonic implications for subduction zones. J. Geophys. Res..

[38] Watanabe, T, Shirasugi, Y, Yano, H. & Michibayashi, K. *Seismic velocity in antigorite-bearing serpentinite mylonites.* **360**, Geological Society: London, 2011.

[39] Pera E, Mainprice D, Burlini L (2003). Anisotropic seismic properties of the upper mantle beneath the Torre Alfina area (Northern Apennines, Central Italy). Tectonophysics.

[40] Shao T (2014). Antigorite‐induced seismic anisotropy and implications for deformation in subduction zones and the Tibetan Plateau. J. Geophys. Res..

[41] Deschamps F, Godard M, Guillot S, Hattori K (2013). Geochemistry of subduction zone serpentinites: a review. Lithos.

[42] Rouméjon S, Cannat M (2014). Serpentinization of mantle‐derived peridotites at mid‐ocean ridges: mesh texture development in the context of tectonic exhumation. Geochem. Geophy. Geosyst..

[43] Lafay R (2013). High-pressure serpentinites, a trap-and-release system controlled by metamorphic conditions: example from the Piedmont zone of the western Alps. Chem. Geol..

[44] Malusà MG (2018). Active carbon sequestration in the Alpine mantle wedge and implications for long-term climate trends. Sci. Rep..

[45] Malusà MG (2015). Contrasting styles of (U) HP rock exhumation along the Cenozoic Adria‐Europe plate boundary (Western Alps, Calabria, Corsica). Geochem. Geophy. Geosyst..

[46] Liao J (2018). Divergent plate motion drives rapid exhumation of (ultra) high pressure rocks. Earth Planet. Sci. Lett..

[47] Bodin T, Yuan H, Romanowicz B (2014). Inversion of receiver functions without deconvolution-application to the Indian craton. Geophys. J. Int..

[48] Sambridge M, Gallagher K, Jackson A, Rickwood P (2006). Trans-dimensional inverse problems, model comparison and the evidence. Geophys. J. Int..

[49] Pilia S (2015). Evidence of micro-continent entrainment during crustal accretion. Sci. Rep..

[50] Young MK, Rawlinson N, Bodin T (2013). Transdimensional inversion of ambient seismic noise for 3D shear velocity structure of the Tasmanian crust. Geophysics.

[51] Green PJ (1995). Reversible jump Markov chain Monte Carlo computation and Bayesian model determination. Biometrika.

[52] Thouvenot F, Paul A, Sénéchal G, Hirn A, Nicolich R (1990). ECORS-CROP wide-angle reflection seismics: constraints on deep interfaces beneath the Alps. Mém. Soc. Géol. France.

[53] Christensen NI (2004). Serpentinites, peridotites, and seismology. Int. Geol. Rev..

[54] Yuan, H. & Dueker, K. Upper mantle tomographic Vp and Vs images of the Rocky Mountains in Wyoming, Colorado and New Mexico: evidence for thick, laterally heterogeneous lithosphere, in: (eds Randy, G. & Karlstrom, K.E.), *The Rocky Mountain Region—an Evolving Lithosphere: Tectonics, Geochemistry, and Geophysics*. 329–345 (American Geophysical Union, Washington, DC, 2005).

[55] Park J (1996). Surface waves in layered anisotropic structures. Geophys. J. Int..

[56] Kern H, Jin Z, Gao S, Popp T, Xu Z (2002). Physical properties of ultrahigh-pressure metamorphic rocks from the Sulu terrain, eastern central China: implications for the seismic structure at the Donghai (CCSD) drilling site. Tectonophysics.

[57] Evans BW (2004). The serpentinite multisystem revisited: chrysotile is metastable. Int. Geol. Rev..

[58] Hilairet N, Daniel I, Reynard B (2006). Equation of state of antigorite, stability field of serpentines, and seismicity in subduction zones. Geophy. Res. Lett..

