# Author Correction: Evidence for a serpentinized plate interface favouring continental subduction

**DOI:** 10.1038/s41467-020-17767-4

**Published:** 2020-07-28

**Authors:** Liang Zhao, Marco G. Malusà, Huaiyu Yuan, Anne Paul, Stéphane Guillot, Yang Lu, Laurent Stehly, Stefano Solarino, Elena Eva, Gang Lu, Thomas Bodin, CIFALPS Group, Liang Zhao, Marco G. Malusà, Anne Paul, Stéphane Guillot, Stefano Solarino, Elena Eva, Gang Lu, AlpArray Working Group, Anne Paul, Stefano Solarino

**Affiliations:** 1 State Key Laboratory of Lithospheric Evolution, Institute of Geology and Geophysics, Chinese Academy of Sciences, Beijing, China; 2 Department of Earth and Environmental Sciences, University of Milano-Bicocca, Milan, Italy; 3 Istituto Nazionale di Geofisica e Vulcanologia, ONT, Genova, Italy; 4 ARC Centre of Excellence for Core to Crust Fluids Systems, Department of Earth and Environmental Sciences, Macquarie University, North Ryde, Australia; 5 Centre for Exploration Targeting, University of Western Australia, Perth, Australia; 6 Geological Survey of Western Australia, Perth, Australia; 7 Univ. Grenoble Alpes, Univ. Savoie Mont Blanc, CNRS, IRD, IFSTTAR, ISTerre, 38000 Grenoble, France; 8 Univ. Lyon, Universite Lyon 1, Ens de Lyon, CNRS, UMR 5276 LGL-TPE, F-69622 Villeurbanne, France

**Keywords:** Geodynamics, Seismology, Tectonics

Correction to: *Nature Communications* 10.1038/s41467-020-15904-7, published online 1 May 2020.

The original version of this article contained an error in Figure 1, in which the heading of panel (c) was mislabelled as ‘40 km’. The correct heading of panel (c) is ‘50 km’. The caption of Figure 1 also contained the same error. Both the panel heading and caption of Figure 1 have now been corrected in the PDF and HTML versions of the article.

