# Supplementary material for: Evidence for a serpentinized plate interface favouring continental subduction
Source: Nat Commun. 2020 May 1;11:2171. doi: 10.1038/s41467-020-15904-7 (PMC7195360; doi:10.1038/s41467-020-15904-7)
Supplement: Supplementary file 1 — Supplementary Information [file 41467_2020_15904_MOESM1_ESM.pdf]

SUPPLEMENTARY INFORMATION

Evidence for a serpentinized plate interface favouring continental subduction

Liang Zhao[1*], Marco G. Malusà[2,3*], Huaiyu Yuan[4,5,6*], Anne Paul[7], Stéphane Guillot[7], Yang Lu[7], Laurent Stehly[7], Stefano Solarino[3], Elena Eva[3], Gang Lu[1], Thomas Bodin[8], CIFALPS group & AlpArray Working Group

[1]State Key Laboratory of Lithospheric Evolution, Institute of Geology and Geophysics, Chinese Academy of Sciences, Beijing, China.

[2]Department of Earth and Environmental Sciences, University of Milano-Bicocca, Milan, Italy.

[3]Istituto Nazionale di Geofisica e Vulcanologia, ONT, Genova, Italy.

[4]ARC Centre of Excellence for Core to Crust Fluids Systems, Department of Earth and Planetary Sciences, Macquarie University, North Ryde, Australia.

[5]Centre for Exploration Targeting, University of Western Australia, Perth, Australia

[6]Geological Survey of Western Australia, Perth Australia

[7]Univ. Grenoble Alpes, Univ. Savoie Mont Blanc, CNRS, IRD, IFSTTAR, ISTerre, 38000 Grenoble, France.

[8]Univ Lyon, Universite Lyon 1, Ens de Lyon, CNRS, UMR 5276 LGL-TPE, F-69622 Villeurbanne, France

[*]Correspondence and requests for materials should be addressed to L.Z. (email: zhaoliang@mail.iggcas.ac.cn), M.G.M. (email: marco.malusa@unimib.it) or H.Y. (email: huaiyu.yuan@mq.edu.au)

Supplementary Information includes:

Supplementary Note

Supplementary Note 1: Details of Rayleigh wave dispersion dataset

Supplementary Note 2: Full list of people contributing to the AlpArray seismic network

Supplementary Figures

Supplementary Figures 1 to 20

Supplementary References

Supplementary files

Supplementary Data 1: A txt file (titled Supplementary Data1.txt) storing dispersion dataset for inversion (by Yang Lu, Laurent Stehly, Anne Paul)

Description to the data file: The file contains local Rayleigh wave group velocity dispersion curves extracted from 2-D tomographic maps at discrete periods for the CIFALPS area (longitude: 4.0-9.5°E; latitude: 43.5-47.0°N). The first and second values of each line are respectively latitude and longitude. The rest values are corresponding group velocity at periods of 5, 7, 9, 11, 13, 15, 17, 19, 21, 23, 25, 28, 31, 34, 37, 40, 43, 46, 49, 52, 55, 60, 65, 70, 75, 80, 85, 90, 95, 100, 110, 120, 130, 140, 150 seconds.

Supplementary Note 1: Details of Rayleigh wave dispersion dataset

The dataset used in this study comes directly from an ambient-noise surface wave tomography (ANT) study of the European crust and uppermost mantle[1]. The ANT study[1] incorporated data of dense arrays of temporary stations in the greater Alpine region, e.g., AlpArray with 50-km spacing and CIFALPS[3] with 5- to 10-km spacing and provided an unprecedented coverage of the western Alps. The dispersion data used in this study were a subset of the 2-D Rayleigh wave group velocity dispersion maps of the ANT study[1] at discrete periods for the Western Alps area (longitude: 4.0-9.5°E; latitude: 43.5-47.0°N, at 0.15° spacing; Supplementary Fig. 1). Group velocities are at periods ranging from 5 to 31 s in 2-s increments, 34 to 55 s in 3 s increments, and 55 to 100 s in 5s increments. In order to evaluate the data coverage and quality, Supplementary Fig. 2 shows a map view of path density in the inversion for group velocity; Supplementary Fig. 3 shows the group velocity data of selected periods for illustration purpose; Supplementary Fig. 4 shows the uncertainty assessment of inversion for group velocity maps using jackknifing tests at periods 8, 40 and 125 s; Supplementary Fig. 5 shows histograms of phase travel time misfits, and Supplementary Fig. 6 shows spatial distribution of the phase travel time misfit of the Vs model evaluated from numerical simulation for Rayleigh waves at 10, 15, 25 and 50 s. Supplementary Fig.7 illustrates examples of dispersion curves along three cross-sections along which the velocity models are shown (Fig 2 & Supplementary Figs 9 to 11). For group velocity measurements, dispersion inversion and resolution tests, we refer readers to details[1,2].

Supplementary Note 2: Full list of people contributing to the AlpArray seismic network

We acknowledge the use of data from the AlpArray network, please visit the project homepage http://www.alparray.ethz.ch/en/seismic_network/backbone/data-policy-and-citation/ (last accessed March 16, 2020) for a full list of people contributing to the AlpArray seismic network. The AlpArray seismic network was made possible by the AlpArray Working Group: György HETÉNYI, Rafael ABREU, Ivo ALLEGRETTI, Maria-Theresia APOLONER, Coralie AUBERT, Simon BESANÇON, Maxime BÈS DE BERC, Götz BOKELMANN, Didier BRUNEL, Marco CAPELLO, Martina ČARMAN, Adriano CAVALIERE, Jérôme CHÈZE, Claudio CHIARABBA, John CLINTON, Glenn COUGOULAT, Wayne C. CRAWFORD, Luigia CRISTIANO, Tibor CZIFRA, Ezio D'ALEMA, Stefania DANESI, Romuald DANIEL, Anke DANNOWSKI, Iva DASOVIĆ, Anne DESCHAMPS, Jean-Xavier DESSA, Cécile DOUBRE, Sven EGDORF, ETHZ-SED Electronics Lab, Tomislav FIKET, Kasper FISCHER, Wolfgang FRIEDERICH, Florian FUCHS, Sigward FUNKE, Domenico GIARDINI, Aladino GOVONI, Zoltán GRÁCZER, Gidera GRÖSCHL, Stefan HEIMERS, Ben HEIT, Davorka HERAK, Marijan HERAK, Johann HUBER, Dejan JARIĆ, Petr JEDLIČKA, Yan JIA, Hélène JUND, Edi KISSLING, Stefan KLINGEN, Bernhard KLOTZ, Petr KOLÍNSKÝ, Heidrun KOPP, Michael KORN, Josef KOTEK, Lothar KÜHNE, Krešo KUK, Dietrich LANGE, Jürgen LOOS, Sara LOVATI, Deny MALENGROS, Lucia MARGHERITI, Christophe MARON, Xavier MARTIN, Marco MASSA, Francesco MAZZARINI, Thomas MEIER, Laurent MÉTRAL, Irene MOLINARI, Milena MORETTI, Anna NARDI, Jurij PAHOR, Anne PAUL, Catherine PÉQUEGNAT, Daniel PETERSEN, Damiano PESARESI, Davide PICCININI, Claudia PIROMALLO, Thomas PLENEFISCH, Jaroslava PLOMEROVÁ, Silvia PONDRELLI,

Snježan PREVOLNIK, Roman RACINE, Marc RÉGNIER, Miriam REISS, Joachim RITTER, Georg RÜMPKER, Simone SALIMBENI, Marco SANTULIN, Werner SCHERER, Sven SCHIPPKUS, Detlef SCHULTE-KORTNACK, Vesna ŠIPKA, Stefano SOLARINO, Daniele SPALLAROSSA, Kathrin SPIEKER, Josip STIPČEVIĆ, Angelo STROLLO, Bálint SÜLE, Gyöngyvér SZANYI, Eszter SZŰCS, Christine THOMAS, Martin THORWART, Frederik TILMANN, Stefan UEDING, Massimiliano VALLOCCHIA, Luděk VECSEY, René VOIGT, Joachim WASSERMANN, Zoltán WÉBER, Christian WEIDLE, Viktor WESZTERGOM, Gauthier WEYLAND, Stefan WIEMER, Felix WOLF, David WOLYNIEC, Thomas ZIEKE, Mladen ŽIVČIĆ, Helena ŽLEBČÍKOVÁ.

Supplementary Figures

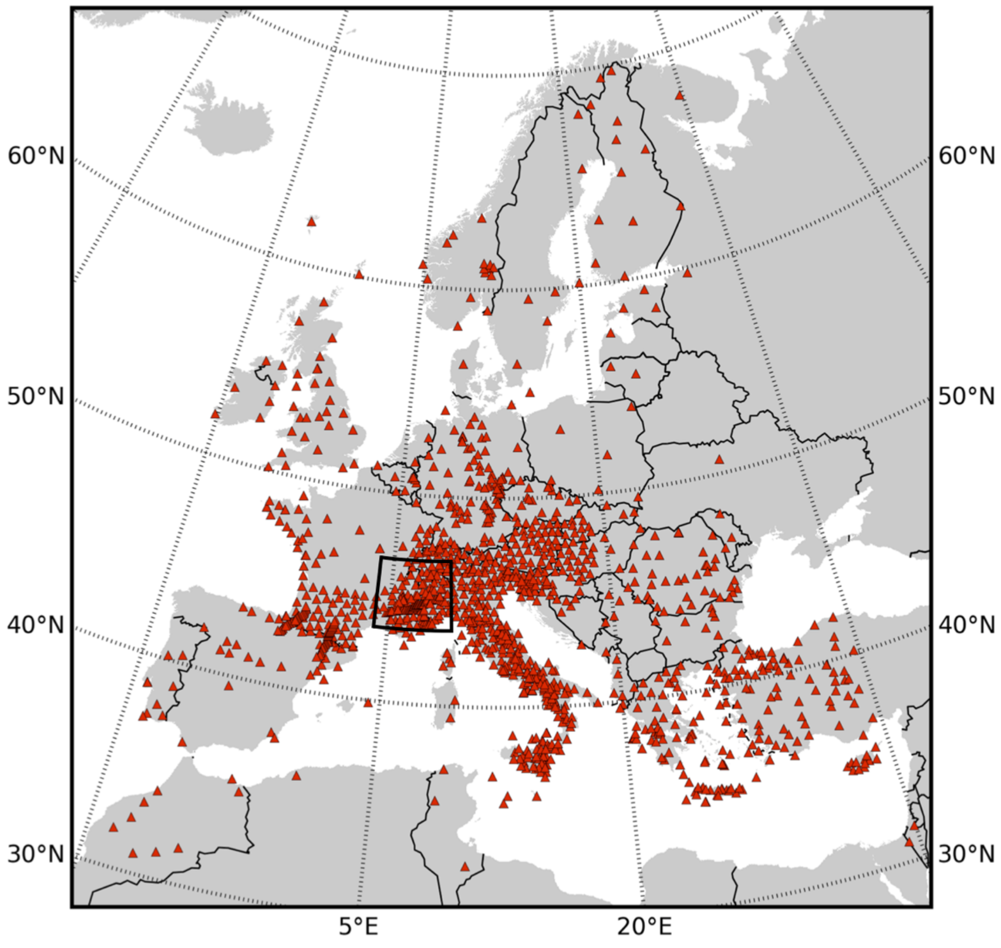

Supplementary Fig. 1. Map of the stations to estimate group velocity dispersion data of Rayleigh waves from ambient noise records[1]. The black box indicates the study region of the present work.

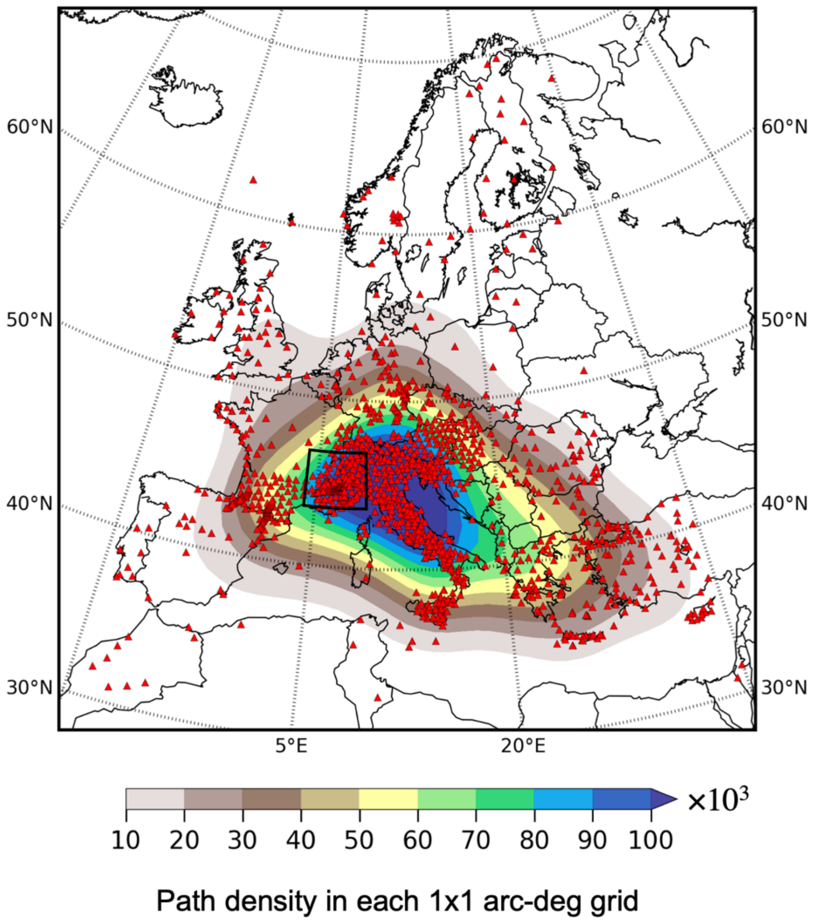

Supplementary Fig. 2. Map of path density in the inversion for group velocity maps[1], measured as the number of paths in each 1° × 1° cell[2]. The black box indicates the study region of the present work. The number of hits is larger than 90,000 per 1°×1° cell over most of the study area except the northwest corner.

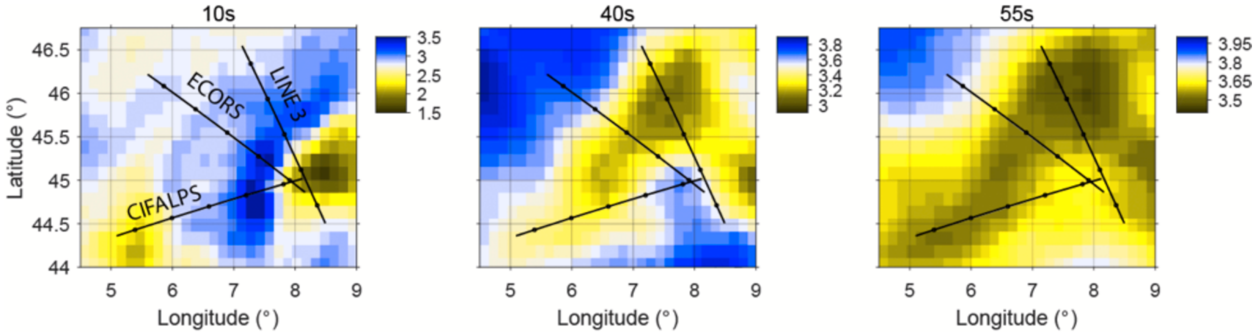

Supplementary Fig. 3. Rayleigh wave group velocity maps Vg (km s^{-1}) at 3 periods, 10s, 40s, and 55s. The three black lines are the locations of cross-sections where inverted velocity structure is shown.

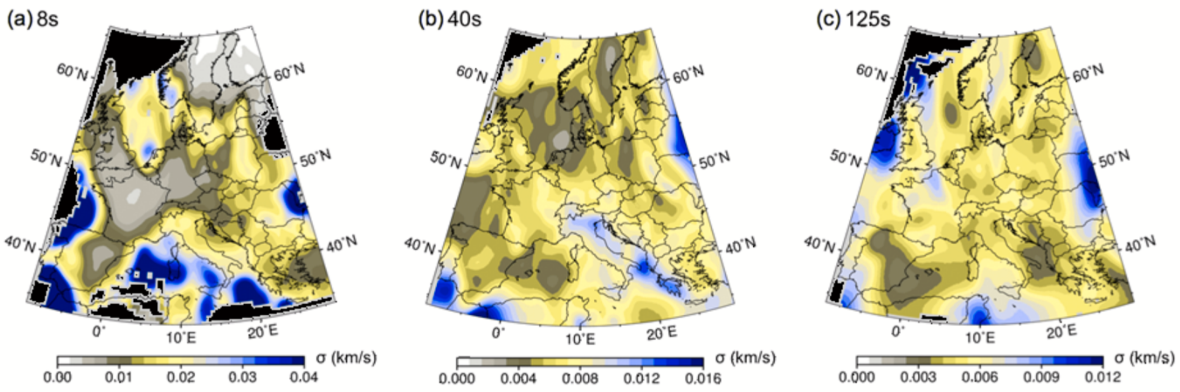

Supplementary Fig. 4. Uncertainty assessment of inversion for group velocity maps using jackknifing tests at periods 8, 40 and 125 s (modified from Fig. S4 in Supplementary Information of Lu et al., 2018[1]). Arbitrarily selected 80% of the original data are used to invert for group velocity maps. This procedure is iterated 30 times. Standard deviations of the ensemble of results are plotted to document the inversion uncertainty. At these 3 periods, and more generally in the entire period band 5-100s used in the present work, the standard deviation is smaller than 0.015 km s^{-1} in most of the study area (black box in Supplementary Figs.1 and 2).

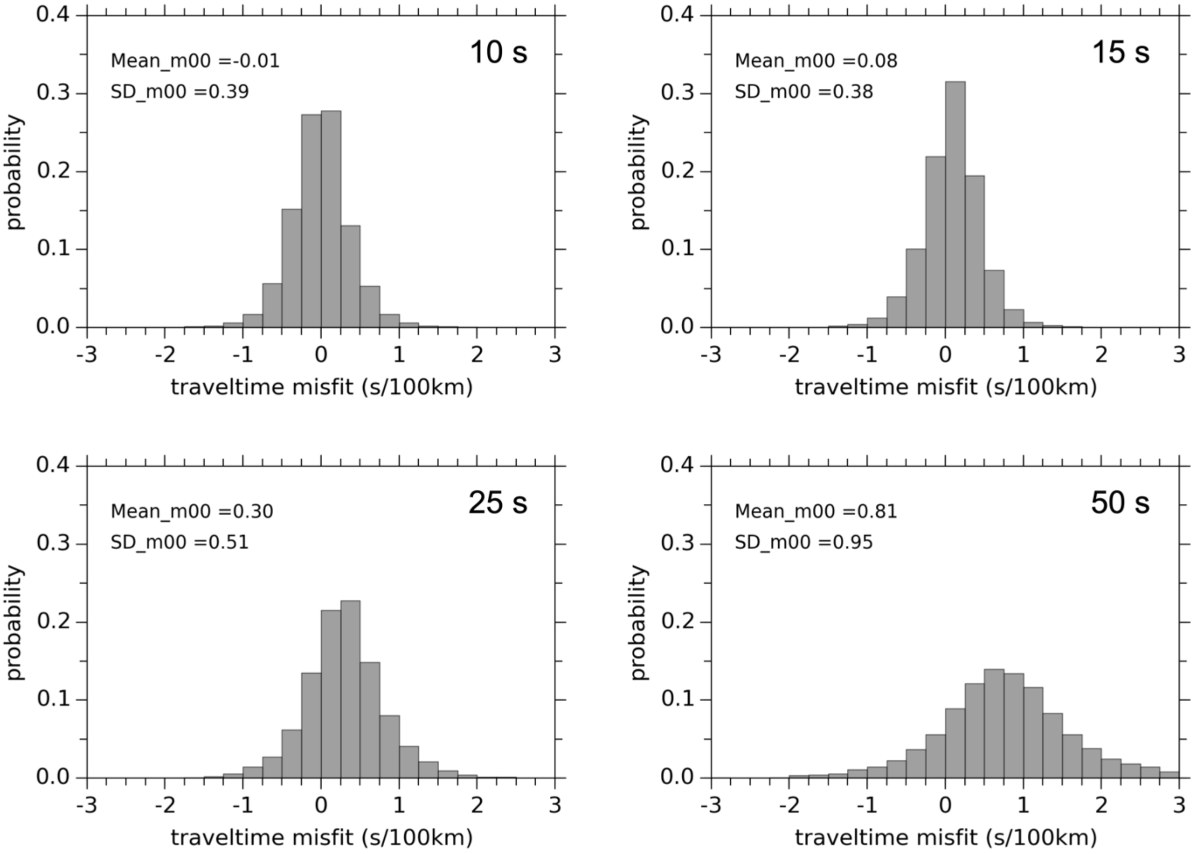

Supplementary Fig. 5. Histograms of phase travel time misfits, defined as the frequency-dependent phase travel time differences between observed waveforms (Rayleigh waves from ambient-noise cross-correlations) and synthetic waveforms computed in the 3-D Vs model of the Alpine region[1,2]. Misfit refers to the travel time misfit for waves propagating 100 km. Mean refers to the mean misfit, while SD refers to the standard deviation. At 25s period, a standard deviation of 0.51s per 100km corresponds to an uncertainty on the phase velocity of 1.5% or 0.045 km s^{-1}.

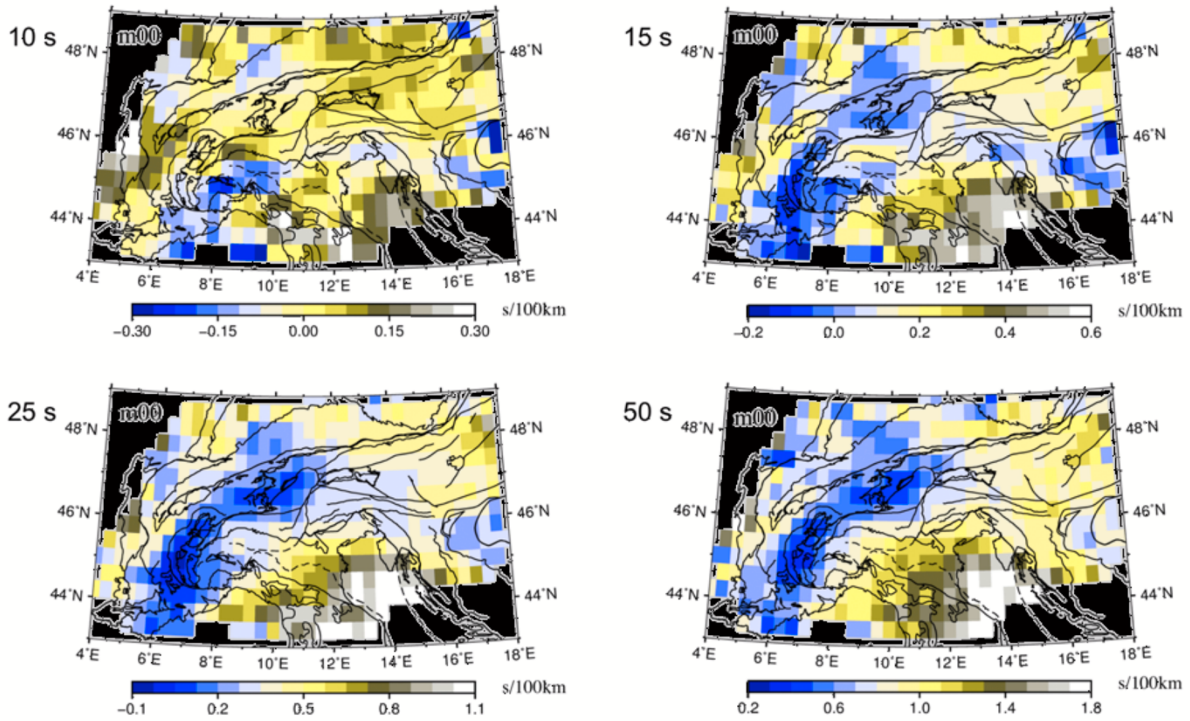

Supplementary Fig. 6. Spatial distribution of the travel time misfit of the Vs model evaluated from numerical simulation for Rayleigh waves at 10, 15, 25 and 50 s (see caption of Supplementary Fig. 5 for the definition of misfit) [2]. At each period, we discretize the study region with a mesh of square cells of size 0.5°×0.5°. For each cell, we calculate the mean value of misfit for all paths crossing the cell assuming ray paths are great-circles. We only display cells crossed by more than 10 paths. The black lines in each map correspond to the geological and tectonic boundaries of the generalized tectonic map of the Alps. In most of the study region of the present work, the misfit at 25s period is smaller than 0.5s per 100 km, which corresponds to an uncertainty on the phase velocity of 1.5% or 0.044 km s^{-1}. This value confirms for the study area the uncertainty estimates made from the misfit histograms of Supplementary Fig. 5 that were computed for the entire Alpine region.

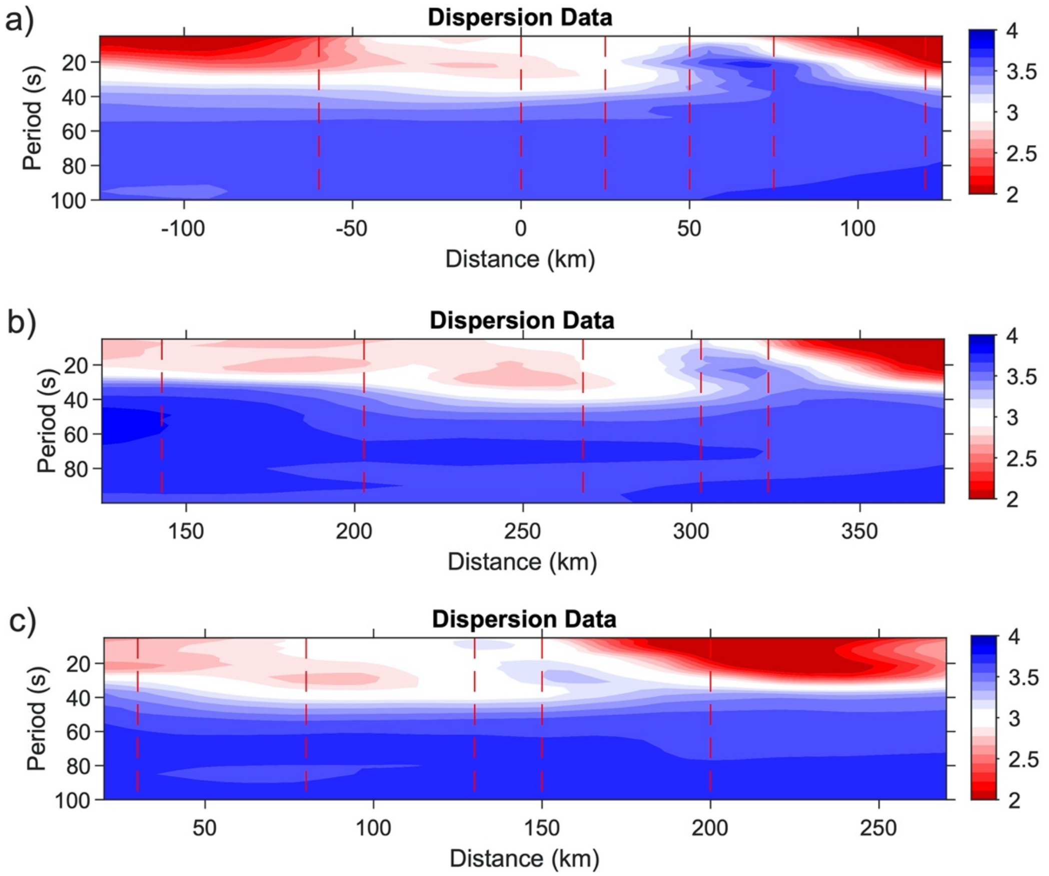

Supplementary Fig. 7. Rayleigh wave group velocities Vg (km s^{-1}) along the 3 cross-sections. **a,** CIFALPS, **b,** ECORS-CROP, and **c,** Line 3. Locations of the 3 profiles are shown in Supplementary Fig.3. Red dashed lines indicate the locations of 1D profiles shown in Fig. 2 and Supplementary Fig. 9-11.

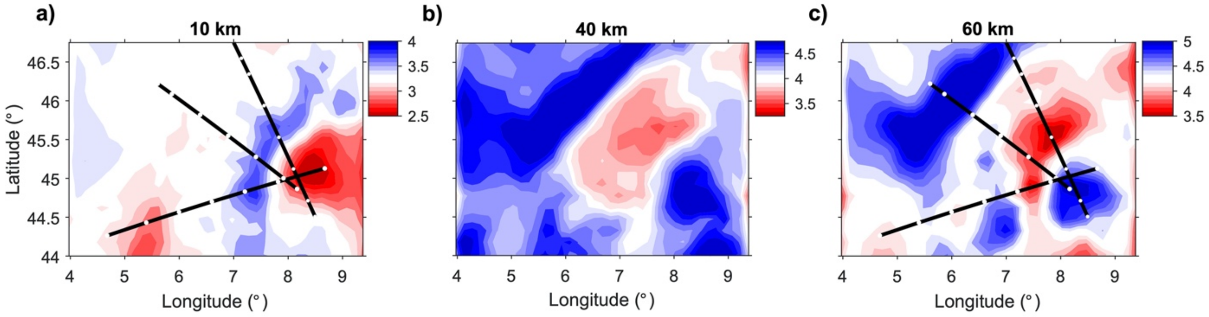

Supplementary Fig. 8. Map views of the inverted shear-wave velocity model at 10-, 40- and 60-km depth. The locations of the 3 cross-sections are labelled in each map. Note the high-velocity structure (in blue) at 10-km depth that corresponds to the Ivrea body along the CIFALPS line between 7 and 7.5° longitude, and the low velocity zone (in orange and red) at 40- and 60-km depth that is sampled by the 3 cross-sections.

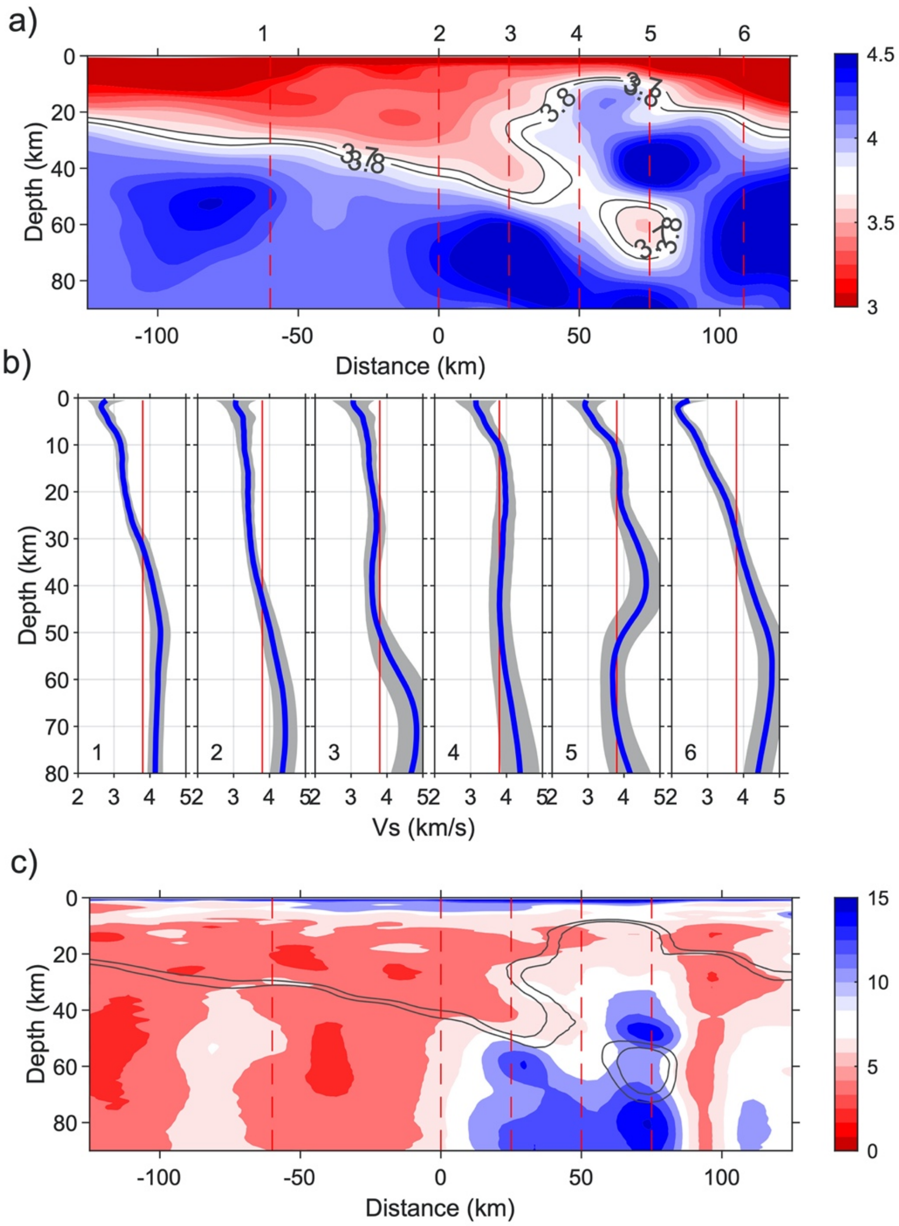

Supplementary Fig. 9. Inversion results along the CIFALPS cross-section. **a,** Absolute shear-wave velocity with respect to depth. The thin black lines indicate the 3.7 and 3.8 km s^{-1} velocity contours. **b,** Depth-velocity profiles at selected locations (labelled in a). The red vertical line indicates the uniform 3.8 km s^{-1} starting model. **c,** Model error (%) approximated by the 1-standard deviation region calculated using the 7.2-million searched models. Note the low velocity region (≤ 3.8 km s^{-1}) at ~30-50 km, ~40-50 km and 50-70 km in depth-velocity profiles 3, 4, and 5 respectively.

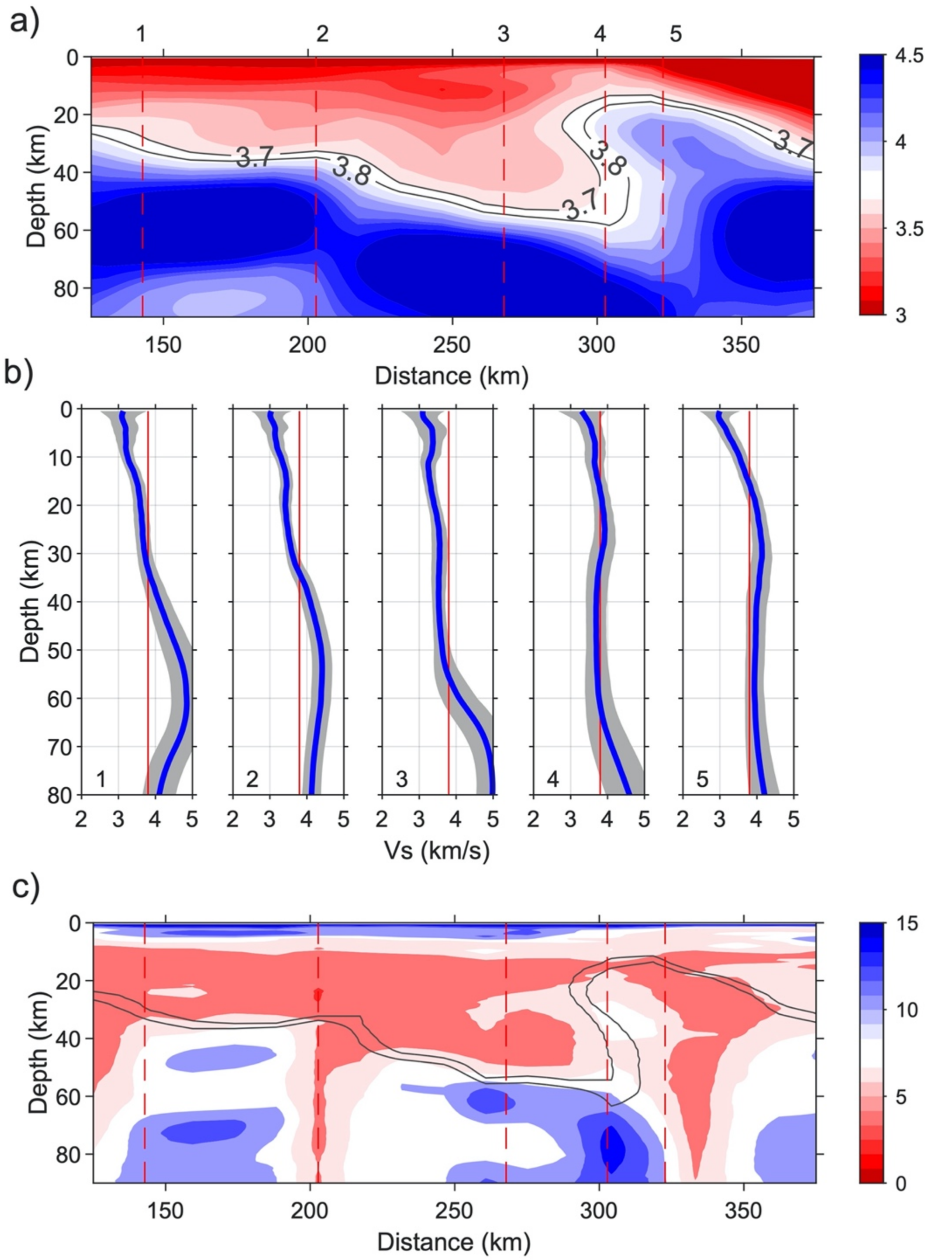

Supplementary Fig. 10. Inversion results along the ECORS-CROP cross-section. **a,** Absolute shear-wave velocity with respect to depth. The thin black lines indicate the 3.7- and 3.8 km s^{-1} velocity contours. The cross-section location follows that in the ambient-noise tomography study[1]. **b,** Depth-velocity profiles at selected locations (labelled in a). The red vertical line indicates the uniform 3.8 km s^{-1} starting model. **c,** Model error (%) approximated by the 1-standard deviation region calculated using the 7.2-million searched models. Note the low velocity region between depth-velocity profiles 3 (30-55 km) and 4 (30-65 km).

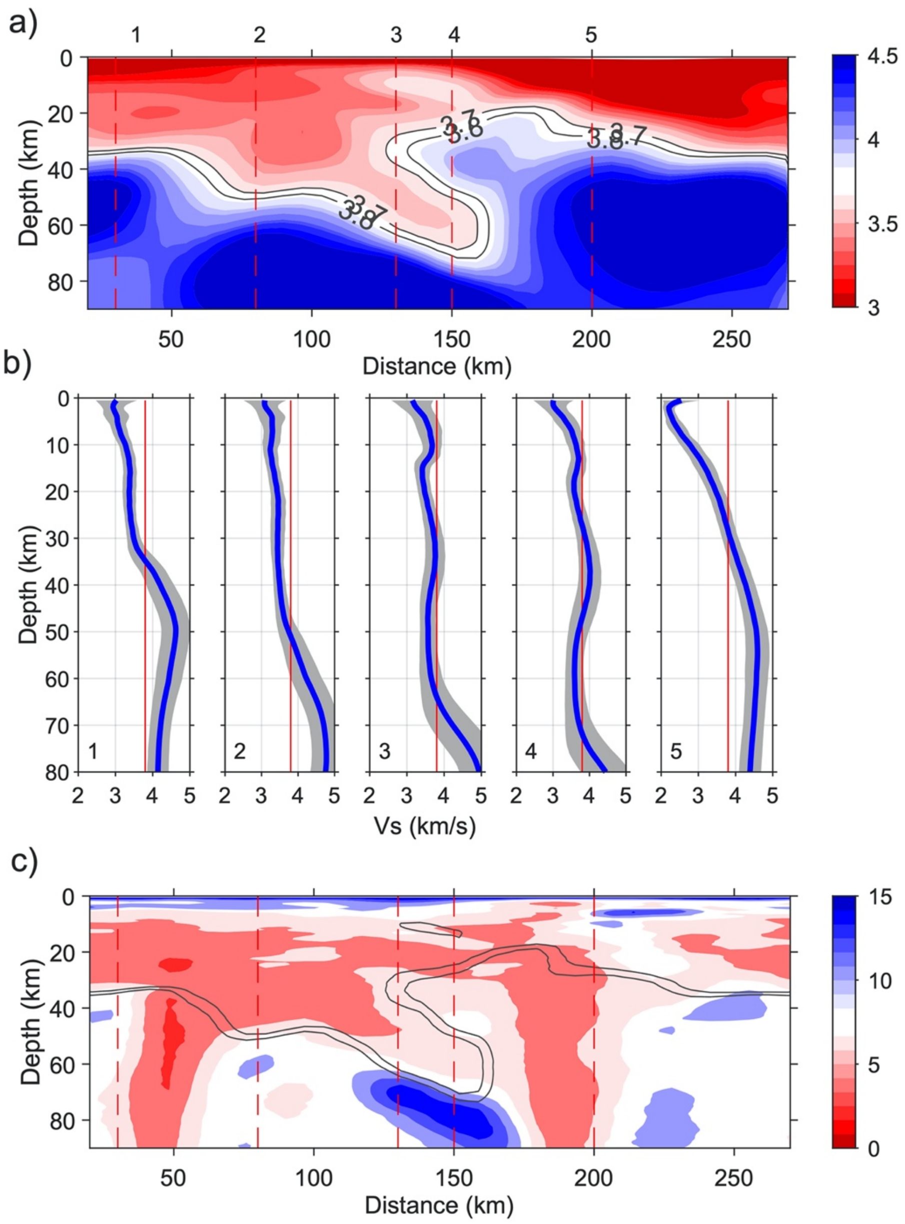

Supplementary Fig. 11. Inversion results along Line 3. **a,** Absolute velocity with respect to depth. The thin black lines indicate the 3.7- and 3.8 km s^{-1} velocity contours. Location of the line is shown in Supplementary Fig. 8. **b,** Depth-velocity profiles at selected locations (labelled in a). The red vertical line indicates the uniform 3.8 km s^{-1} starting model. **c,** Model error (%) approximated by the 1-standard deviation region calculated using the 7.2-million searched models. Note the low velocity region in depth-velocity profiles 3 (40-65 km) and 4 (45-70 km).

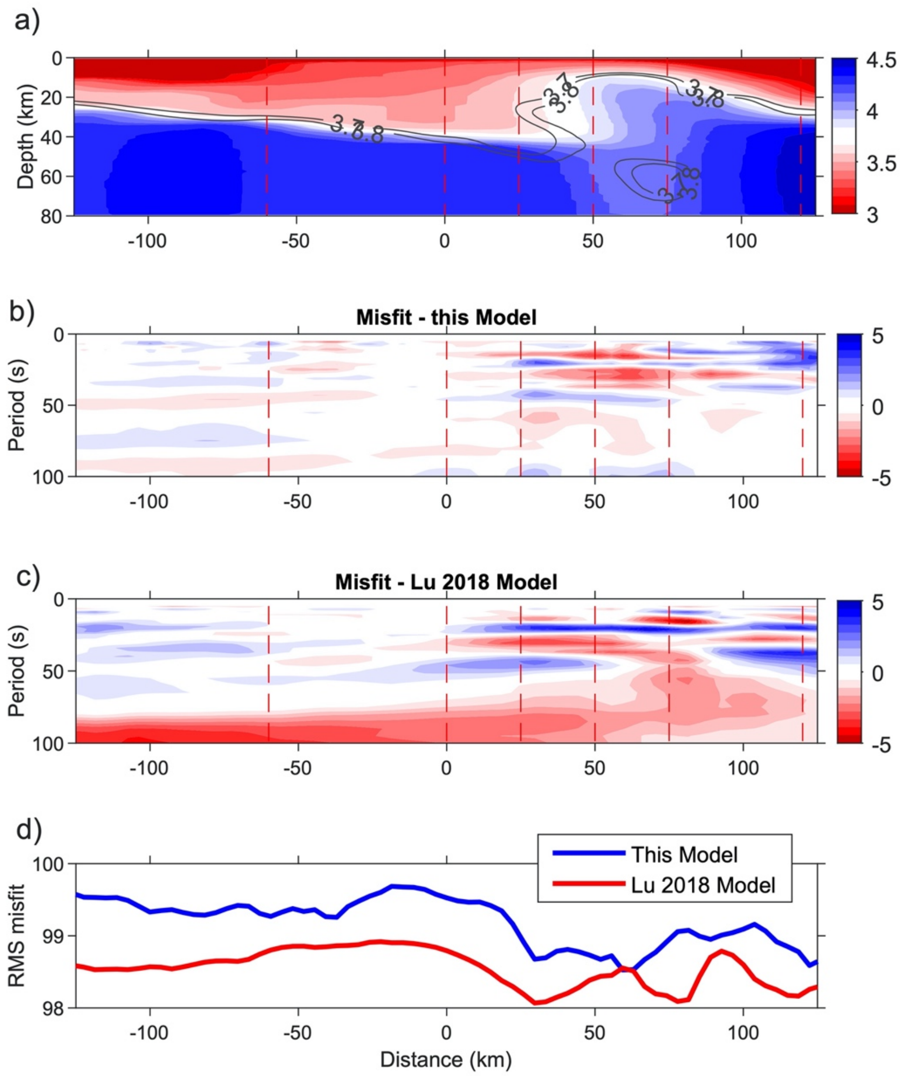

Supplementary Fig. 12. Model comparison for the CIFALPS line. **a**, Model of the ambient-noise tomography (ANT)[1]. The thin black lines show the 3.7- and 3.8 km s^{-1} velocity contours of the new model. **b**, Misfit between data and model prediction from this study (in %). Note that the y-axis is in period. **c**, Misfit between data and the ANT model[1] (in %). **d**, A comparison of variance reduction between data and model predictions along the cross section. Note that a better fit is obtained with the new model across the region of low velocity between 20 and 70 km distance.

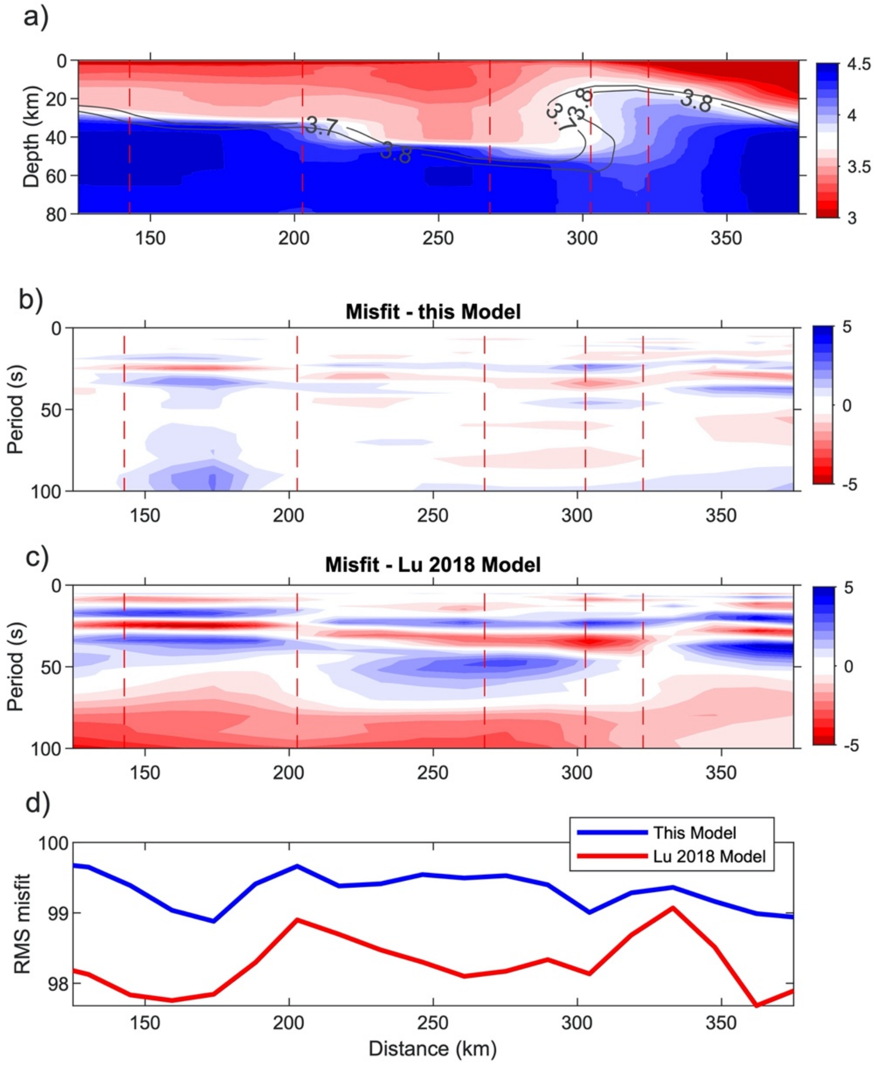

Supplementary Fig. 13. Model comparison for the ECORS-CROP line. **a**, Model of the ambient-noise tomography (ANT) [1]. The thin black lines show the 3.7- and 3.8 km s^{-1} velocity contours of the new model. **b**, Misfit between data and model prediction from this study (in %). Note that the y-axis is in period. **c**, Misfit between data and the ANT model[1] (in %). **d**, A comparison of data variance reduction between data and model predictions along the cross section. Note that a better fit is obtained with the new model across the region of low velocity (~300 km distance).

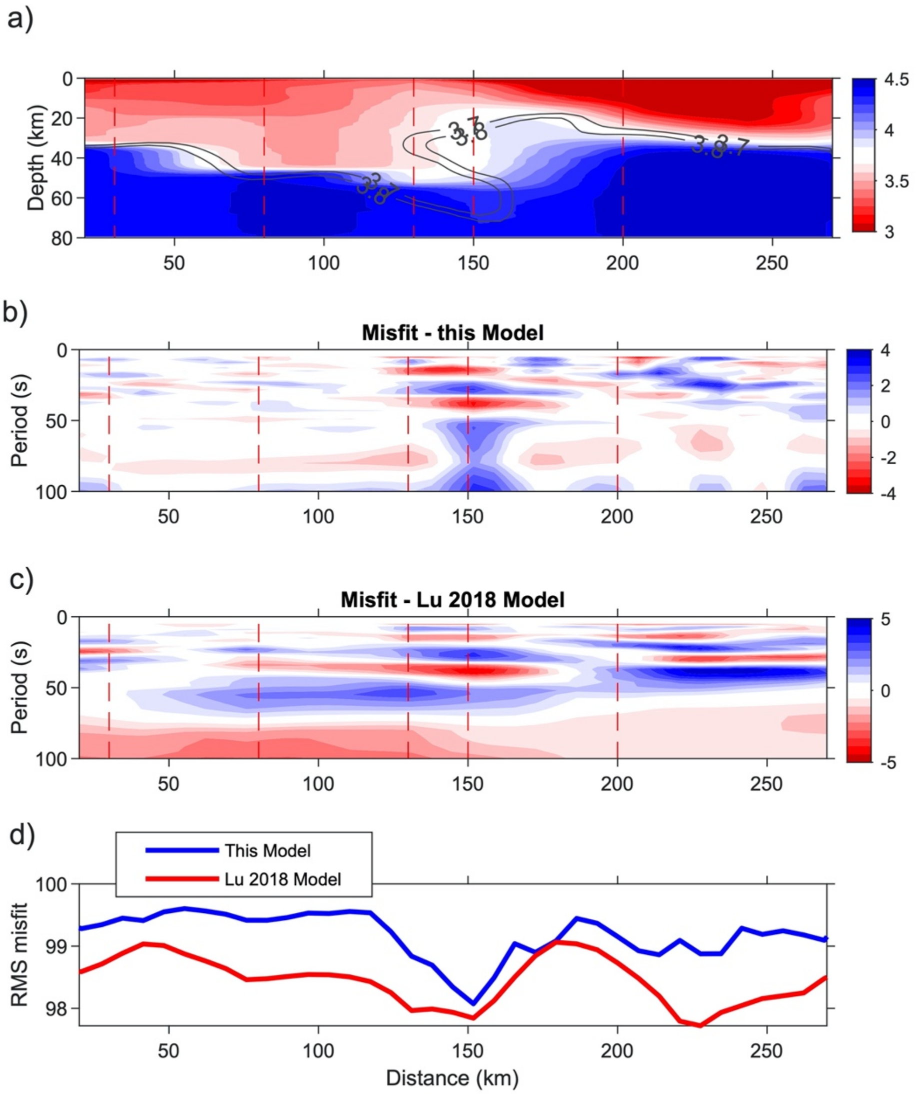

Supplementary Fig. 14. Model comparison for Line 3. **a**, Model of the ambient-noise tomography (ANT)[1]. The thin black lines show the 3.7 and 3.8 km s^{-1} velocity contours of the new model. **b**, Misfit between the data and model prediction from this study (in %). Note that the y-axis is in period. **c**, Misfit between data and the ANT model[1] (in %). **d**, A comparison of data variance reduction between data and model predictions along the cross section. Note that a better fit is obtained with the new model across the region of low velocity (distance 130 to 150 km).

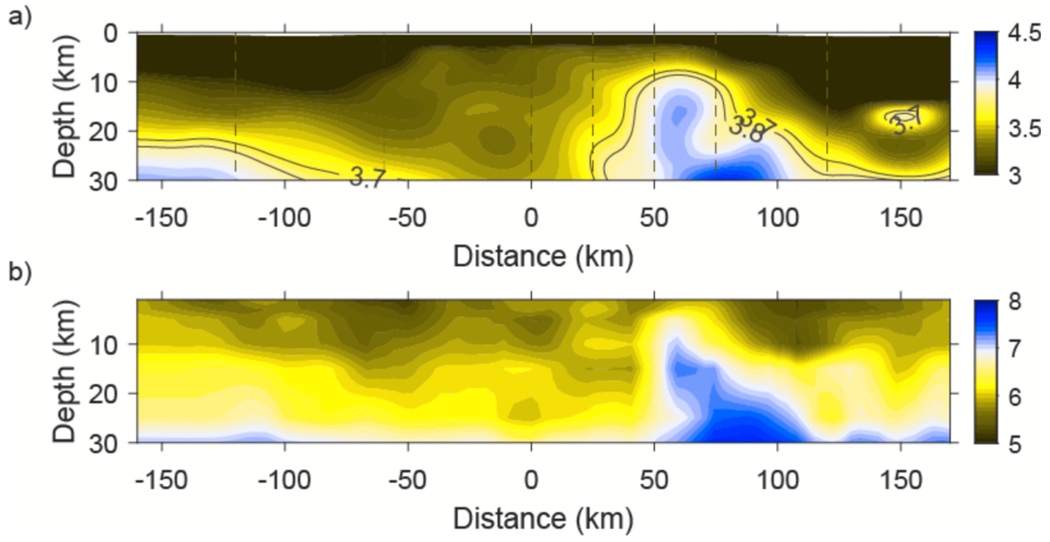

Supplementary Fig. 15. A comparison of the spatial consistency of the Ivrea high velocity body (distance 50 to 100 km) between our Vs model (a) and the Vp model[4] (b) along the CIFALPS profile**.**

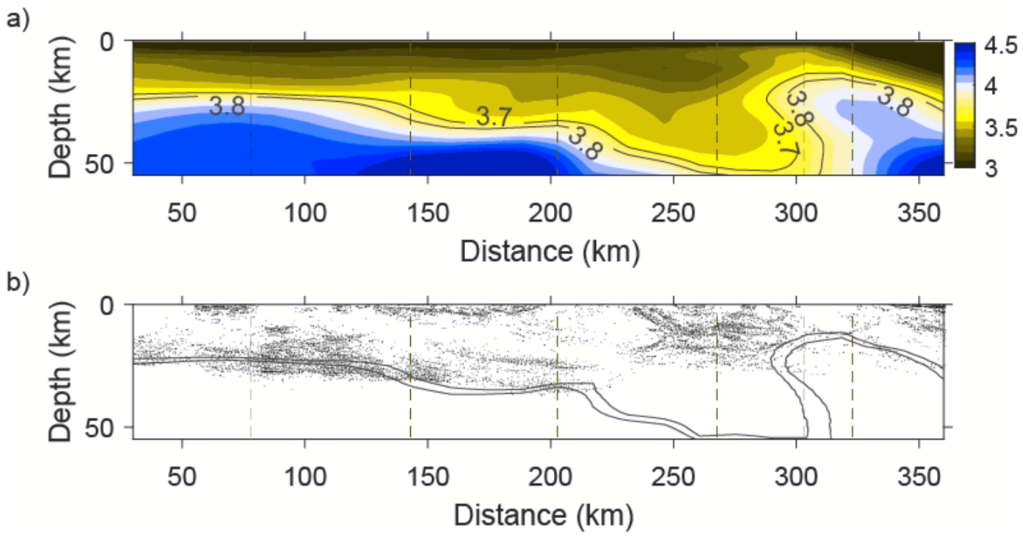

Supplementary Fig. 16. A comparison between our velocity model (a) and the ECORS-CROP deep seismic reflection profile (b). The ECORS-CROP deep seismic reflection image[5].

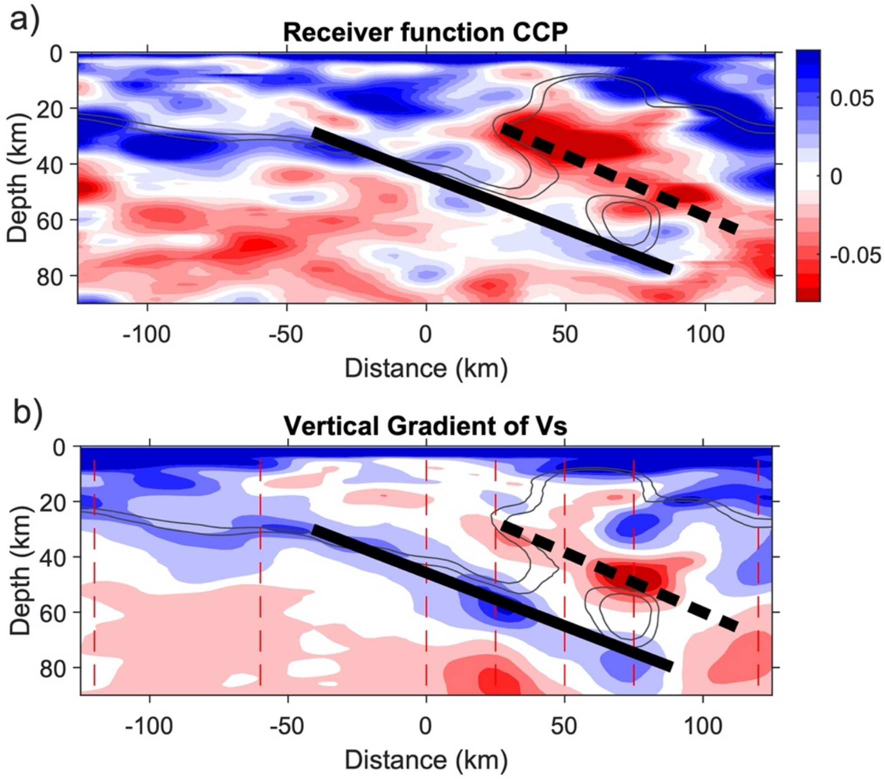

Supplementary Fig. 17. A comparison between **a**, the receiver function Common-Conversion-Point (CCP) stacked model[6], and **b**, the depth gradient of the final Vs model along the CIFALPS profile. Note that **a** and **b** share the same color scale. The thin lines indicate the 3.7 and 3.8 km s^{-1} velocity contours. The dashed thick lines show the negative velocity gradient on top of the low velocity zone described in this study, while the solid thick line shows the bottom of the low velocity zone.

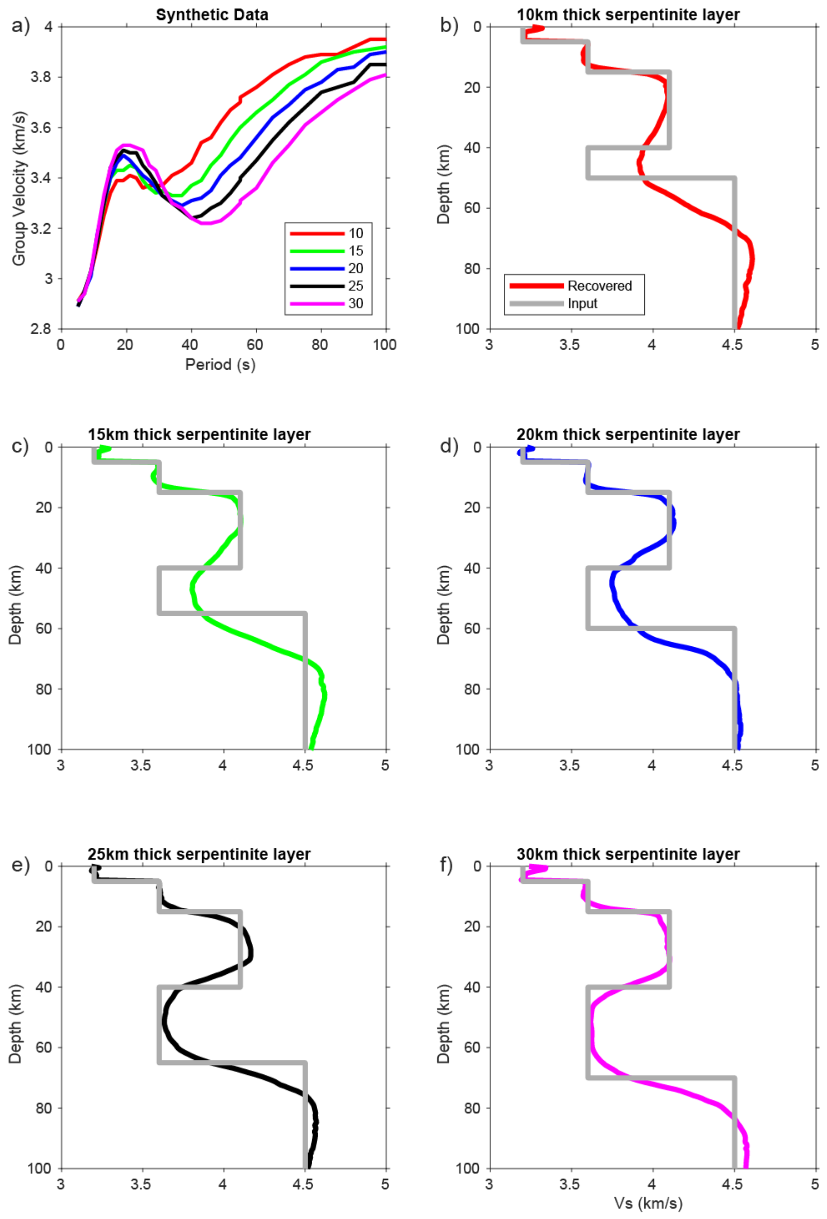

Supplementary Fig. 18. Layer thickness recovery tests. **a**, Group velocity dispersion curves computed using simple layered velocity models over an isotropic half space. The thickness of the slow-velocity layer varies from 10- to 30-km as illustrated by the thick grey line in **b** to **f**. Note that the amplitude of the slow velocity layer is under-estimated if the layer thickness is less than 25km.

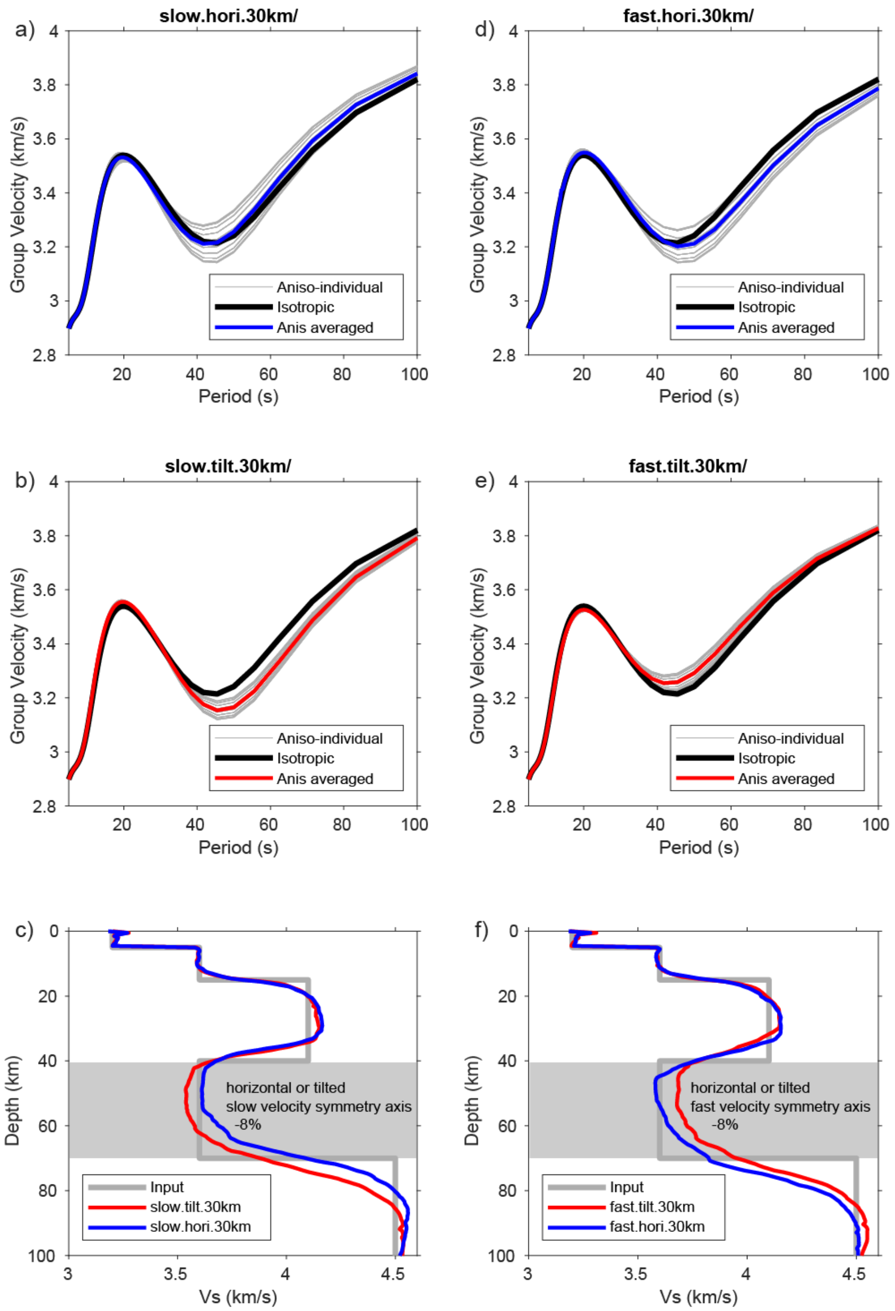

Supplementary Fig. 19. Simple tests to illustrate effects on dispersion curves of a 30-km low velocity anisotropic layer with a horizontal or tilted symmetry axis. **a,** and **b,** Dispersion curves for horizontal and tilted slow velocity symmetry axes; **c,** Results of isotropic transdimensional tomographic inversion. For comparison, **d** to **f** are the same as **a** to **c** but for fast velocity symmetry axes.

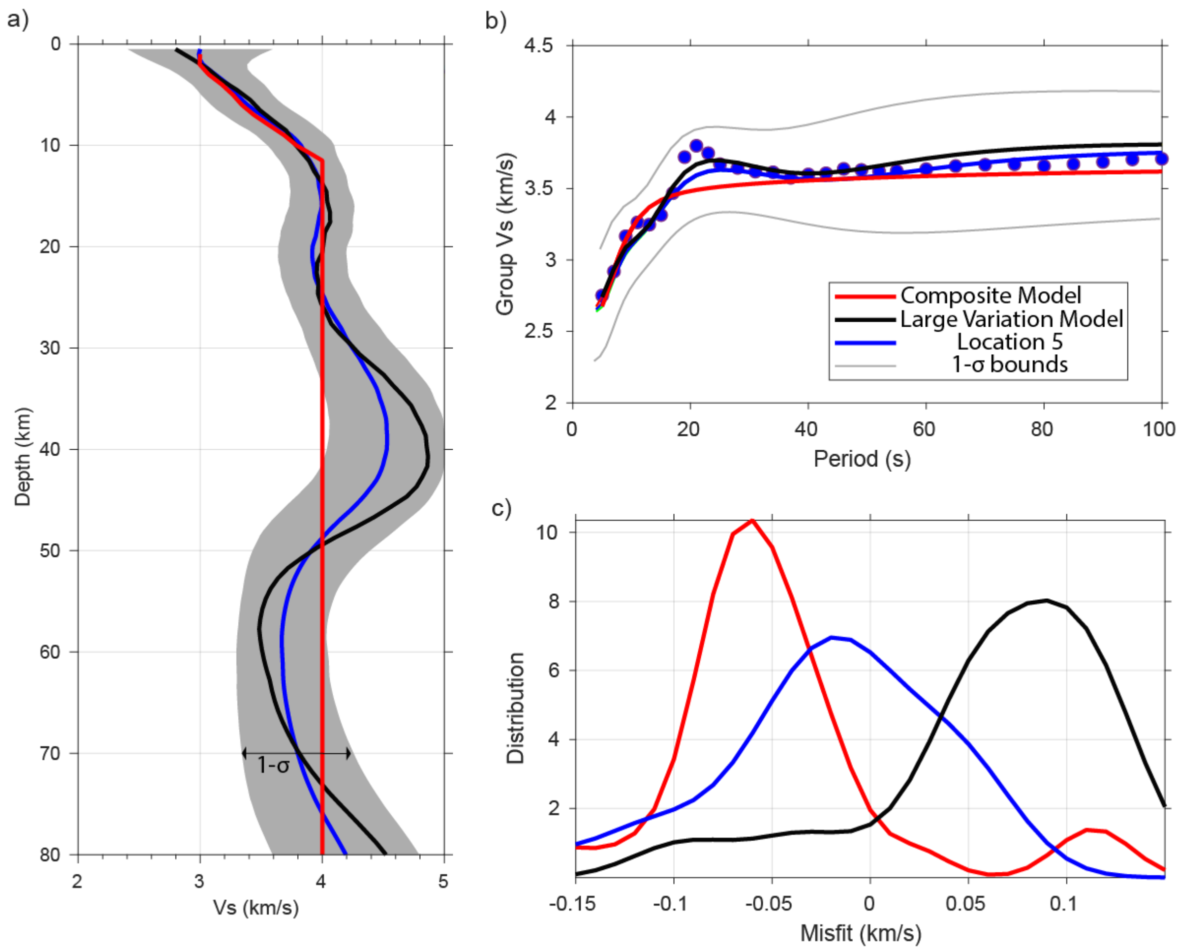

Supplementary Fig. 20. Evaluation of two additional test models for the robustness of the slow-velocity layer. **a**, Velocity models. Blue, final model presented at Location 5 in Figure 2a. The grey area shows the 1-σ model uncertainty. Red, a composite model which is similar to the Blue model from 0 to 10 km but has a homogeneous 4 km s^{-1} velocity from 10km downwards. Black, a model selected from the inversions which has larger velocity amplitudes in the slow velocity zone (50 to 75 km depth) and the high velocity layer above (30-50 km). **b**, Computed dispersions. Blue dots show the data. Velocity models and the 1-sigma bounds are labeled. **c**, Misfit distribution of the three models. Note that the final model (blue) has a distribution whose peak is more centered towards zero and is largely unimodal, so is favored over the test models.

Supplementary References

1. Lu, Y., Stehly, L., Paul, A. & AlpArray Working Group. High-resolution surface wave tomography of the European crust and uppermost mantle from ambient seismic noise. *Geophys. J. Int.* **214**, 1136–1150 (2018).
2. Lu, Y. Tomography of the alpine arc using noise correlations & waveform modelling, Ph-D thesis, Univ. Grenoble Alpes, https://tel.archives-ouvertes.fr/tel-02135198 (2019).
3. Zhao, L., Paul, A., Solarino, S. & RESIF. Seismic network YP: CIFALPS temporary experiment (China-Italy-France Alps seismic transect), RESIF. doi: 10.15778/RESIF.YP2012 (2016).
4. Solarino, S., Malusà, M.G., Eva, E., Guillot, S., Paul, A., Schwartz, S., Zhao, L., Aubert, C., Dumont, T., Pondrelli, S., Salimbeni, S., Wang, Q., Xu, X., Zheng, T. & Zhu, R. Mantle wedge exhumation beneath the Dora-Maira (U)HP dome unravelled by local earthquake tomography (Western Alps). *Lithos* **296**, 623-636 (2018).
5. Thouvenot, F., Paul, A., Sénéchal, G., Hirn, A. & Nicolich, R. ECORS-CROP wide-angle reflection seismics: constraints on deep interfaces beneath the Alps. *Mém. Soc. Géol. Fr.* **156**, 97-106 (1990).
6. Zhao, L., Paul, A., Malusà, M. G., Xu, X., Zheng, T., Solarino, S., Guillot, S., Schwartz, S., Dumont, T., Salimbeni, S., Aubert, C., Pondrelli, S., Wang, Q. & Zhu, R. Continuity of the Alpine slab unraveled by high-resolution P-wave tomography. *J. Geophys. Res.* **121**, 8720-8737 (2016).
