# Supplementary material for: Evidence for a serpentinized plate interface favouring continental subduction
Source: Nat Commun. 2020 May 1;11:2171. doi: 10.1038/s41467-020-15904-7 (PMC7195360; doi:10.1038/s41467-020-15904-7)
Supplement: Supplementary file 2 — Description of Additional Supplementary Files [file 41467_2020_15904_MOESM2_ESM.pdf]

Description of Additional Supplementary Files

File Name: Supplementary Data 1

Description: **A txt file storing dispersion dataset for inversion (by Yang Lu, Laurent Stehly, Anne Paul).** The file contains local Rayleigh wave group velocity dispersion curves extracted from 2-D tomographic maps at discrete periods for the CIFALPS area (longitude: 4.0-9.5°E; latitude: 43.5-47.0°N). The first and second values of each line are respectively latitude and longitude. The rest values are corresponding group velocity at periods of 5, 7, 9, 11, 13, 15, 17, 19, 21, 23, 25, 28, 31, 34, 37, 40, 43, 46, 49, 52, 55, 60, 65, 70, 75, 80, 85, 90, 95, 100, 110, 120, 130, 140, 150 seconds.
